# A comprehensive study based on large-sample multi-omics integration and machine learning to decode mitochondria-associated genes: from digestive tract tumours to gastric cancer

**DOI:** 10.3389/fimmu.2026.1719986

**Published:** 2026-07-06

**Authors:** Wei Zhang, Yu-Lu Tang, Yi-Yang Chen, Ya Shang, Hong-Bo Mo, Jie Huang, Yu-Feng Li, Ze-Hua Liu, Ge-Li Tan, Yan-Kun Ning, Guo-Qiang Chen, Jing-Wen Ling, Lei Wang, Jia-Shu Jiang, Jia-Yuan Luo, Gang Chen

**Affiliations:** 1 Department of Pathology, The First Affiliated Hospital of Guangxi Medical University, Nanning, Guangxi Zhuang Autonomous Region, China; 2 International Cooperation and External Exchange Department, The First Affiliated Hospital of Guangxi Medical University, Nanning, Guangxi Zhuang Autonomous Region, China

**Keywords:** digestive tract tumour, immune exclusion, LACTB2, miRNA early diagnosis, prognosis

## Abstract

**Background:**

Mitochondria-related genes play a crucial role in driving tumour cell progression, but little is known about their molecular mechanisms and biological pathways. This study conducted a comprehensive analysis of the mitochondrial key gene LACTB2 in digestive tract tumours and explored a novel early blood-based diagnostic model for gastric cancer (GC).

**Methods:**

This study analyzed LACTB2 expression, biological pathways, and immune regulation in a large cohort of 10,581 samples. IHC staining was performed using 236 internal GC samples. Multi-omics data were integrated for comprehensive analysis of LACTB2 in GC. A combination of extensive clinical samples and multiple machine learning models enabled the construction of prognostic and blood-based diagnostic models (n = 14,219).

**Results:**

LACTB2 overexpression is associated with clinical metastasis and the activation of pro-cancer pathways, and LACTB2 may mediate immune suppression and immune evasion through various methods. There is a significant transcriptional regulatory network upstream of LACTB2. LACTB2 overexpression may drive malignant transformation of GC epithelial cells through pro-cancer metabolic signalling networks, and the related mechanisms were spatially validated. Dysregulated expression of LACTB2 can affect the prognosis of GC patients, and Afatinib and Ulixertinib may play a significant role in targeting LACTB2 in the treatment of GC patients. An excellent early blood diagnostic model for GC was constructed based on the upstream miRNA of LACTB2.

**Conclusion:**

Our study provides new insights into the differential expression and pathogenesis of LACTB2 in digestive tract tumours, particularly its prognostic and diagnostic value in GC.

## 1 Introduction

Digestive tract tumours (including colon adenocarcinoma (COAD), rectum adenocarcinoma (READ), gastric cancer (GC), and esophageal carcinoma (ESCA)) pose a severe threat to human life and health, collectively accounting for a leading share of global cancer incidence and mortality (1, 2). Among these, due to its close association with lifestyle and dietary habits, GC is particularly prominent among digestive tract tumours (3). Global data from 2022 indicates that the incidence and mortality rates of GC account for 4.9% and 6.8% of the total global cancer cases and deaths, respectively (both ranked fifth), with stomach adenocarcinoma(STAD) being the most significant subtype of GC, comprising as much as 90-95% (1, 4). Due to the insidious nature of early symptoms, the five-year survival rate for patients with advanced gastric cancer is only 20–40% (5, 6). Combination therapies (such as immunotherapy combined with targeted therapy or chemotherapy) offer hope for patients with advanced GC (7–9). However, traditional screening methods face challenges in widespread adoption due to their invasive nature or insufficient sensitivity, while emerging liquid biopsy techniques lack reliable biomarkers (10, 11). Consequently, the identification of novel diagnostic and therapeutic targets for GC is an urgent priority.

In recent years, research has found that mitochondrial metabolic reprogramming can drive tumour progression through energy supply, anti-apoptosis, and metabolite toxicity, becoming a new direction for anti-cancer therapy (12–14). Among them, mitochondrial RNA metabolism regulation is a key link in maintaining mitochondrial function, and lactamase beta 2 (LACTB2), as a mitochondrial RNA endoribonuclease, regulates metabolic gene expression by cleaving mRNA/tRNA (15, 16). LACTB2 may promote tumour development by affecting mitochondrial function and disrupting the cell cycle process, and it has been found to be associated with poor prognosis in various cancers (17). Among these, in nasopharyngeal carcinoma, LACTB2 can regulate the PINK1/Parkin axis to enhance the radioresistance of tumour cells, and in colorectal cancer, LACTB2 can disrupt the tumour-suppressive function of NCOA2 (18, 19). However, since its function was first explicitly investigated in 2021, the role of LACTB2 in gastrointestinal tumours, particularly GC, remains poorly documented in systematic reports (19). Therefore, this study aims to systematically elucidate the molecular characteristics of LACTB2 in the progression of gastrointestinal tumours and its potential for early diagnosis (20).

## 2 Materials and methods

### 2.1 Analysis of LACTB2 mRNA expression in digestive tract tumours

This study included clinical samples of LACTB2 mRNA from a variety of digestive tract tumours obtained from ArrayExpress, The Cancer Genome Atlas (TCGA) and the Gene Expression Omnibus (GEO). The inclusion exclusion steps for digestive tract tumours–related microarray and RNA-seq data were shown in **Supplementary Figure S1**. The Genotype-Tissue Expression (GTEx) project contains data from a wide range of healthy human tissues (21). Ultimately, this study included 169 transcriptomic datasets comprising 10,581 digestive tract tumour tissue samples (**Table 1**). After processing and normalising the included datasets using the “limma” package and log_2_(x+1) transformation, we merged datasets from the same experimental platform and eliminated the batch effect using the “SVA” package (22, 23). Information about patients’ clinical characteristics and prognoses can be downloaded directly from TCGA.

**Table 1:** Basic information about the high-throughput sequencing datasets included in this study.

Category	Dataset	Platform	Country	Year	Cancer samples	Non-cancer samples
CRC tissue mRNA	GSE24514	GPL96	Finland	2018	34	15
	GSE49355	GPL96	France	2018	20	18
	GSE68468	GPL96	USA	2018	374	14
	GSE77953	GPL96	USA	2018	17	13
	GSE110223	GPL96	Greece	2019	13	13
	GSE21815	GPL6480	Japan	2019	132	9
	GSE35279	GPL6480	Japan	2019	74	5
	GSE141174	GPL6104	Sweden	2020	3	3
	GSE10972	GPL6104	Singapore	2013	24	24
	GSE3629	GPL570	Japan	2019	68	53
	GSE4107	GPL570	Singapore	2019	12	10
	GSE4183	GPL570	Hungary	2019	15	8
	GSE5206	GPL570	USA	2019	100	5
	GSE9348	GPL570	Singapore	2019	70	12
	GSE13471	GPL570	USA	2019	4	4
	GSE15960	GPL570	Hungary	2019	6	6
	GSE21510	GPL570	Japan	2019	123	25
	GSE23878	GPL570	Saudi Arabia	2019	35	24
	GSE32323	GPL570	Japan	2019	17	17
	GSE33113	GPL570	Netherlands	2019	90	6
	GSE37364	GPL570	Hungary	2019	14	38
	GSE41328	GPL570	USA	2019	10	10
	GSE62932	GPL570	USA	2019	64	4
	GSE110224	GPL570	Greece	2019	17	17
	GSE20842	GPL4133	Germany	2018	65	65
	GSE35982	GPL4133	China	2018	8	8
	GSE15781	GPL2986	Norway	2016	13	10
	GSE25071	GPL2986	Norway	2019	46	4
	GSE115261	GPL23126	Singapore	2020	10	10
	GSE139814	GPL23126	China	2020	6	6
	GSE113513	GPL15207	China	2022	14	14
	GSE81558	GPL15207	Spain	2018	23	9
	GSE106582	GPL10558	Germany	2018	77	117
	GSE54986	GPL10558	China	2018	6	6
	GSE75548	GPL10558	China	2018	6	6
	GSE62321	GPL97	France	2018	20	18
	GSE41011	GPL6254	China	2015	19	12
	GSE47063	GPL6102	UK	2015	14	4
	GSE156355	GPL21185	China	2021	6	6
	GSE126092	GPL21047	China	2020	10	10
	GSE184093	GPL20115	China	2021	9	9
	GSE100179	GPL17586	Hungary	2022	20	20
	GSE44076	GPL13667	Spain	2023	98	148
	GSE87211	GPL13497	USA	2018	203	160
	GSE103512	GPL13158	USA	2021	57	12
	TCGA_GTEx_CRC	RNA-seq	——	——	458	349
ESCA tissue mRNA	GSE47763	GPL10558	Germany	2018	3	0
	GSE57130	GPL10558	Germany	2018	3	3
	GSE72873	GPL10558	Australia	2018	48	17
	GSE93352	GPL10558	France	2018	3	0
	GSE45168	GPL13497	China	2018	5	5
	GSE97558	GPL13497	China	2019	3	0
	GSE103356	GPL16791	USA	2021	0	6
	GSE111011	GPL16791	USA	2019	7	7
	GSE113341	GPL16791	USA	2019	0	10
	GSE113777	GPL16791	China	2019	0	1
	GSE116272	GPL16791	USA	2019	0	4
	GSE156651	GPL16791	USA	2024	0	4
	GSE74553	GPL17692	USA	2017	52	8
	GSE77563	GPL17692	USA	2019	0	40
	GSE142556	GPL20301	USA	2021	2	0
	GSE149609	GPL20301	USA	2020	17	10
	GSE124514	GPL20795	China	2020	2	0
	GSE128913	GPL20795	China	2019	3	0
	GSE128914	GPL20795	China	2020	3	0
	GSE164158	GPL20795	China	2021	8	8
	GSE49292	GPL5175	USA	2019	0	3
	GSE65013	GPL5175	USA	2019	0	6
	GSE75241	GPL5175	Brazil	2020	15	15
	GSE75243	GPL5175	Brazil	2019	2	0
	GSE100942	GPL570	China	2021	4	10
	GSE11373	GPL570	China	2019	1	0
	GSE13378	GPL570	Ireland	2019	8	0
	GSE146808	GPL570	China	2020	3	0
	GSE148247	GPL570	Netherlands	2019	0	6
	GSE161533	GPL570	China	2020	28	112
	GSE17351	GPL570	USA	2019	5	10
	GSE17353	GPL570	USA	2019	0	8
	GSE19472	GPL570	USA	2019	0	4
	GSE26886	GPL570	Germany	2019	30	38
	GSE27424	GPL570	USA	2019	0	12
	GSE32701	GPL570	Japan	2019	20	0
	GSE33810	GPL570	China	2019	2	2
	GSE3526	GPL570	USA	2022	0	8
	GSE44021	GPL570	USA	2019	6	12
	GSE35975	GPL570	China	2019	1	0
	GSE42363	GPL570	USA	2019	14	0
	GSE45670	GPL570	China	2019	0	20
	GSE63941	GPL570	Japan	2019	22	0
	GSE64894	GPL570	USA	2019	0	3
	GSE67508	GPL570	China	2019	8	0
	GSE69925	GPL570	Japan	2019	274	0
	GSE7307	GPL570	USA	2019	0	8
	GSE77861	GPL570	USA	2019	7	14
	GSE7964	GPL570	USA	2019	1	0
	GSE86013	GPL570	Singapore	2019	2	0
	GSE9974	GPL570	USA	2019	3	0
	GSE20347	GPL571	USA	2018	17	17
	GSE29001	GPL571	USA	2018	21	24
	GSE36223	GPL571	Poland	2018	0	23
	GSE38129	GPL571	USA	2018	30	30
	GSE39491	GPL571	USA	2018	0	40
	GSE44021	GPL571	USA	2022	73	73
	GSE53892	GPL571	Denmark	2018	3	0
	GSE100843	GPL6244	USA	2021	0	18
	GSE34619	GPL6244	UK	2018	0	8
	GSE36725	GPL6244	USA	2018	0	5
	GSE92396	GPL6244	USA	2018	12	10
	GSE13083	GPL96	USA	2018	0	14
	GSE1420	GPL96	USA	2018	8	16
	GSE23400	GPL96	USA	2019	53	106
	GSE37200	GPL96	USA	2021	15	0
	GSE44021	GPL96	USA	2022	34	68
	GSE52138	GPL96	USA	2018	1	4
	GSE70409	GPL13287	China	2016	17	17
	GSE45350	GPL13607	Canada	2018	4	4
	GSE53624	GPL18109	China	2020	119	119
	GSE28302	GPL2507	Australia	2012	23	9
	GSE13898	GPL6102	USA	2013	75	28
	GSE119436	GPL9052	China	2019	4	4
	TCGA_GTEx_ESCA	RNA-seq	——	——	161	594
GC tissue mRNA	E-MTAB-3732	ArrayExpress_Agilent	UK	2022	138	20
	E-TABM-424	ArrayExpress_Agilent	Korea	2022	1	1
	E-MEXP-3479	ArrayExpress_Agilent	Denmark	2022	3	0
	GSE13911	GPL570	Italy	2019	38	31
	GSE15459	GPL570	Switzerland	2019	196	0
	GSE19826	GPL570	China	2019	12	15
	GSE34942	GPL570	Singapore	2019	56	0
	GSE35809	GPL570	Singapore	2019	70	0
	GSE38749	GPL570	Brazil	2019	15	0
	GSE42252	GPL570	Japan	2019	5	0
	GSE54129	GPL570	China	2019	111	21
	GSE57303	GPL570	China	2019	70	0
	GSE62254	GPL570	USA	2019	300	0
	GSE64951	GPL570	USA	2020	63	31
	GSE66222	GPL570	USA	2019	0	100
	GSE79973	GPL570	China	2019	10	10
	GSE206329	GPL24676	China	2022	6	3
	GSE236522	GPL24676	China	2024	33	0
	GSE194261	GPL17077	Japan	2022	10	10
	GSE130823	GPL17077	China	2020	16	47
	GSE136755	GPL17077	Japan	2019	43	0
	GSE84787	GPL17077	China	2020	10	10
	GSE113255	GPL18573	South Korea	2020	130	10
	GSE147043	GPL18573	South Korea	2020	8	0
	GSE152415	GPL18573	USA	2021	47	0
	GSE165211	GPL18573	Israel	2021	9	0
	GSE33335	GPL5175	China	2019	25	25
	GSE63089	GPL5175	China	2019	45	45
	GSE29998	GPL6947	Singapore	2018	50	49
	GSE26942	GPL6947	USA	2019	205	12
	GSE29272	GPL96	USA	2018	134	134
	GSE52138	GPL96	USA	2018	25	13
	GSE208099	GPL21185	Japan	2023	16	16
	GSE220917	GPL28102	Germany	2023	18	5
	GSE191275	GPL20301	China	2023	20	10
	GSE51575	GPL13607	South Korea	2018	26	26
	GSE112369	GPL15207	Japan	2018	36	6
	GSE109476	GPL24530	China	2019	5	5
	GSE103236	GPL4133	Romania	2021	10	9
	GSE116312	GPL6244	Mexico	2019	3	10
	GSE13861	GPL6884	USA	2020	71	19
	GSE20143	GPL9365	India	2013	5	2
	GSE158662	GPL22755	China	2020	3	3
	GSE30727	GPL5188	South Korea	2014	30	30
	TCGA_GTEx_STAD	——	——	——	415	394
GC blood miRNA	GSE211692	GPL21263	Japan	2023	1418	5643
	GSE164174	GPL21263	Japan	2021	1417	1417
	GSE112264	GPL21263	Japan	2019	50	41
	GSE124158	GPL21263	Japan	2019	30	275
	GSE113486	GPL21263	Japan	2019	40	100
	GSE106817	GPL21263	Japan	2025	115	2759
	GSE59856	GPL18941	Japan	2018	50	150

CRC, colorectal cancer; ESCA, esophageal cancer; GC, gastric cancer; STAD, stomach adenocarcinoma.

The mitochondrial gene set can be obtained from MitoCarta 3.0 (https://www.broadinstitute.org/). The protein interaction network from STRING (https://cn.string-db.org/) was imported into Cytoscape (v.3.8.2), and the hub genes in the protein-protein interaction (PPI) network were identified using the “CytoHubba” (v0.1) plugin. The Depmap Portal provides LACTB2 Chronos scores associated with CRISPR screens for various digestive tract tumour cell lines (n = 210) (24). Protein level data can be obtained directly from the Human Protein Atlas (THPA) (https://www.proteinatlas.org/) and UALCAN (https://ualcan.path.uab.edu/index.html). OpenGPS allows the prediction of protein localisation and the acquisition of the corresponding nuclear and protein images (25). To further elucidate the transcriptional regulatory network of LACTB2, this study used the Cistrome Data Browser (http://cistrome.org/db/#/) to predict regulatory elements upstream of the transcription start site (TSS), and integrated multiple ChIP-seq and ATAC-seq experimental data to validate the initial predictions (26). Chromloops, as a novel platform for integrating multi-source chromatin conformation capture data (including PLAC-Seq, ChIA-PET and HiChIP), provides a standardised repository for the resolution of 3D genomic interactions and the construction of dynamic regulatory networks (27).

### 2.2 Biological pathway analysis and immunocorrelation analysis of LACTB2 in digestive tract tumours

In this study, LACTB2 was screened for co-expressed genes in each digestive tract tumour dataset using Pearson’s coefficient *r*. If the Pearson correlation coefficient *r* ≥ 0.3, *p* < 0.05 and a gene met this condition in at least three datasets, this indicated that the gene may be co-expressed with LACTB2. Highly expressed genes in digestive tract tumour tissues were intersected with LACTB2 co-expressed genes, and the intersection results were defined as highly expressed co-expression genes (HECEGs) of LACTB2. KEGG, GO and Reactome enrichment analyses were performed using the “clusterProfiler’ package and the “ReactomePA’ package (28–30). TCGA-based cancer samples were divided into high-expression (>=50%) and low-expression (<50%) groups based on the expression level of LACTB2. Next, the c2.cp.kegg.v7.4.symbols.gmt subset was downloaded from the Molecular Signatures Database and analysed by GSEA software (v.3.0).

In addition, we performed six different tumour microenvironment (TME) quantification and treatment response prediction based on “IOBR’, and analysed immunophenotype features using multi-omics data (CIBERSORT, EPIC, MCPcounter, ESTIMATE, TIMER and QUANTIseq) (31). Further, based on the set of 28 immune signature genes proposed by Charoentong et al. we quantified sample-specific immune cell infiltration scores using ssGSEA (32, 33).

### 2.3 GC clinical sample in-house IHC experiment

In this study, immunohistochemical (IHC) experiments were performed to detect the expression level of the LACTB2 protein in GC tissues and to evaluate its association with GC. To avoid non-specific staining interference, the experimental groups were normal control, disease group, positive control and negative control. GC tissue samples were obtained from Guilin Fanpu Biotech of Guangxi (Guilin Fanpu Biotech of Guangxi, China, a subsidiary wholly owned by Pantomics, Inc), and tissue microarrays were made (approved by the Ethics Committee of the First Affiliated Hospital of Guangxi Medical University, batch NO. 2022-KT-GuiWei-070). All operations in this study were performed in accordance with the Declaration of Helsinki (revised 2013). All patients explicitly agreed to the study and signed an informed consent form. The tissue samples were fixed in 4% paraformaldehyde at room temperature for 12–24 hours, then subjected to a gradient ethanol dehydration process, followed by paraffin embedding and serial sectioning at a thickness of 4μm. The sections were baked at 60 °C for two hours to enhance adhesion, followed by xylene dewaxing and gradient ethanol rehydration. Antigen retrieval was performed using the heat-mediated antigen retrieval method in sodium citrate buffer (pH 6.0), followed by treatment with 3% hydrogen peroxide for 10 minutes at room temperature to block endogenous peroxidase activity. The primary antibody was selected for incubation in a wet box at 4 °C overnight. The secondary antibody was incubated at room temperature for 30 minutes and washed with a PBS washing solution at the end of each incubation (Abcam, ab154267; dilution ratio 1:500). The negative control was established by conducting the experiment using PBS in place of the specific primary antibody, to confirm the specificity of the staining. Finally, the DAB chromogenic solution was used to make the positive signal clear. Real-time monitoring was performed under a microscope until a brownish-yellow signal appeared. The nuclei of the cells were then re-stained with haematoxylin for 30 seconds, differentiated with 1% hydrochloric acid ethanol for five seconds, immersed in 0.1% ammonia for five seconds to return to blue, and sealed with neutral resin after gradient ethanol dehydration. Photographic records were taken using a light microscope. In the samples, brown staining indicates positive antibody expression, whereas blue staining indicates negative expression. The IHC experimental results were scored using the H-score method. Each sample was scored in ten randomly selected fields of view, and the average of these scores was taken as the final score.

### 2.4 scRNA-seq analysis

This study included the GSE163558 dataset for single-cell RNA sequencing (scRNA-seq) analysis. Quality control and downstream analysis of the scRNA-seq data were performed using the “Seurat” package (34, 35). Samples were combined to exclude those with gene expression in fewer than three cells or with intracellular gene content of less than 200. Any low-quality cells with mitochondrial gene content exceeding 20% of the total UMI count were then filtered out. Following dimensionality reduction and cell clustering, as well as a subsequent secondary dimensionality reduction, the “Harmony” package was used for batch data correction (36, 37). Finally, “SingleR” and “CellMarker” were used for cell type annotation (38, 39).

The “inferCNV” package was used to infer copy number variation (CNV) by scRNA-seq and identify malignant cells (40). The “CytoTRACE” and “Monocle3” packages were combined for single-cell pseudotime analysis (41, 42). In scRNA-seq, virtual knockout (KO) of LACTB2 can be performed using the “scTenifoldKnk” package (43). In addition, we used “CellChat” to deconstruct multidimensional communication and resolve cell subpopulation signalling interactions, based on a ligand-receptor (L-R) pair coexpression probability model (44). The “AUCell” and “scMetabolism” packages enable the calculation of AUC values for each cell and assessment of single-cell metabolic activity (45, 46).

### 2.5 Comprehensive spatial transcriptomics analysis

The dataset GSM7990475 and GSM7990480 for GC-associated spatial transcriptomics analysis is available from GEO. The data were read and processed using the “Seurat” package. This was followed by SCT normalisation, data clustering and UMAP downscaling. The spatial areas after clustering were delineated and confirmed by two senior pathologists. The “SPATA2” package was used to analyse CNV in ST data (47). Cell type deconvolution analysis was then performed using the “CARD” package to enable the spatial localisation of cell types (48). Next, spatial trajectory and co-localisation analyses were performed using the “Monocle” and “MistyR” packages (49, 50). Using spatial coordinates, we performed a permutation−based distance test to compare the observed mean minimum distance from immune cells to LACTB2−high spots against a null distribution from 1,000 random permutations. We used the “SpaCET” package to estimate cell lineages and cell-to-cell interactions in the TME (51). Furthermore, the “CellChat” package was used to infer spatially proximal intercellular communication between interacting cell populations from spatially resolved transcriptomics. The “decoupleR” package was used to infer pathway activity (52).

### 2.6 Multiple machine learning combined to develop prognostic models for GC and early diagnostic models for blood-based miRNAs

Four GC prognostic datasets (TCGA, GSE13861, GSE15459, GSE26901) were obtained based on TCGA and GEO and screened for the set of LACTB2 positively related genes associated with poor prognosis. TCGA was used as the training set, while the remaining three datasets were used as the validation set, in order to construct the prognostic model. Ten different machine learning algorithms covering 101 combinations were integrated: stepwise Cox, generalised boosted regression modelling (GBM), random survival forest (RSF), elastic net (Enet), Ridge, CoxBoost, supervised principal components (SuperPC), Lasso, partial least squares regression for Cox (PLSRcox) and survival support vector machine (Survival-SVM). One hundred and one algorithm combinations were integrated using leave-one-out cross-validation (LOOCV). The validation cohort was then cross-validated for all models and Harrell’s concordance index (C-index) was finally calculated for all validation datasets in order to evaluate the models. The next step of prognostic analysis involved time-dependent weight adjustment, nonlinear feature correction and dynamic hierarchical fusion processing on the models’ risk score.

GC blood microRNA (miRNA) datasets can be downloaded from the GEO database. The specific inclusion and exclusion steps are shown in **Supplementary Figure S1**. Seven datasets were finally included (**Table 1**). The blood miRNA data were processed uniformly according to Methods 2.1 in the article. Finally, two cohorts comprising 13,505 GC blood samples were synthesised to participate in constructing the cancer blood miRNA early screening model. We integrated 12 machine learning algorithms, covering 113 combinations, including Lasso, Ridge, Enet, Stepglm, SVM, Glmboost, Lda, Plsglm, Random forest, Gbm, Xgboost and Naivebayes. In the context of cross-validation, one algorithm was used for variable selection and another to construct a classification prediction model. The area under the curve (AUC) of the receiver operating characteristic (ROC) of the model combination was then calculated on an external dataset (or including a training set). Finally, the model’s evaluation results were visualised.

During model selection, we aimed to balance predictive performance with clinical interpretability by prioritising models that achieved high accuracy while requiring fewer features, such as those with the highest C-index or AUC on the independent validation set while maintaining a minimal set of genes or miRNAs. This strategy enhances clinical translation potential while ensuring predictive performance. Kaplan-Meier analyses were performed using the R packages “survival” (v.3.8.3) and “survminer” (v.0.5.0). The “rms” (v.7.0.0) and “rmda” (v.1.6) packages were used to plot Nomograms, decision curve analyses (DCA) and Calibration Curves for evaluating model performance.

### 2.7 Prediction of drugs targeting LACTB2 overexpression and molecular docking

In this study, we selected the top 150 highly and lowly expressed genes (|SMD|> 0, *p* < 0.05) in GC samples to screen for candidate compounds with GC gene expression patterns (53). We integrated GC transcriptome data and *in vitro* drug sensitivity information from GDSC (v.2.0). We then used the “oncoPredict” package to build a cross-platform IC50 prediction model based on ridge regression and ultimately generated drug sensitivity scores (54). The results for GC drug-treated cell lines were obtained from GSE141352.

The PDB format file for LACTB2 (PDB ID: 4AD9) was available on the RCSB PDB database (https://www.rcsb.org/). The SDF structure file for the molecule drug was downloaded via PubChem database (https://pubchem.ncbi.nlm.nih.gov/). Next, molecular docking was performed using CB-Dock2 (https://cadd.labshare.cn/cb-dock2/php/index.php) (55). After docking, the PDB protein-ligand file was downloaded for subsequent analysis and visualised in 2D using Discovery Studio 4.5 Client.

### 2.8 Statistical analysis

In this study, the Wilcoxon rank-sum test and SMD were employed to assess between-group variability. For datasets exhibiting significant heterogeneity (adjudication criteria: heterogeneity index I² > 50% and correlation test *p* ≤ 0.05), the random effects model was favoured for estimating the effect size. The statistical inference criterion was considered significant when the 95% confidence interval for the effect size did not include zero or *p* < 0.05. T-test and ANOVA analyses were used to compare mean differences in clinical characteristics between groups for both two-sample and multi-sample datasets. Model robustness was verified by sensitivity analysis if the Egger’s regression test suggested no significant publication bias (*p* > 0.1). The discriminatory efficacy of cancer versus normal samples was assessed using the AUC based on ROC versus composite subject characteristics (sROC) curves, with an AUC greater than 0.7 being considered a valid discrimination threshold. To ensure the robustness of all bioinformatics findings and control false positives, this study adopted the following strategies: (1) All p-values from differential expression, correlation, and survival analyses underwent false discovery rate correction; (2) Key conclusions were cross-validated using multiple independent computational methods or datasets; (3) Functional enrichment analyses employed the entire gene set from authoritative databases as background.

Heterogeneity tests and analyses of clinical characteristics were performed using the Stata (v.17.0) and SPSS (v.27.0) statistical software packages. All calculations and visualisations were performed using R (v.4.4.3), unless otherwise specified.

## 3 Results

### 3.1 LACTB2 expression was dysregulated in digestive tract tumours

**Figure 1:**
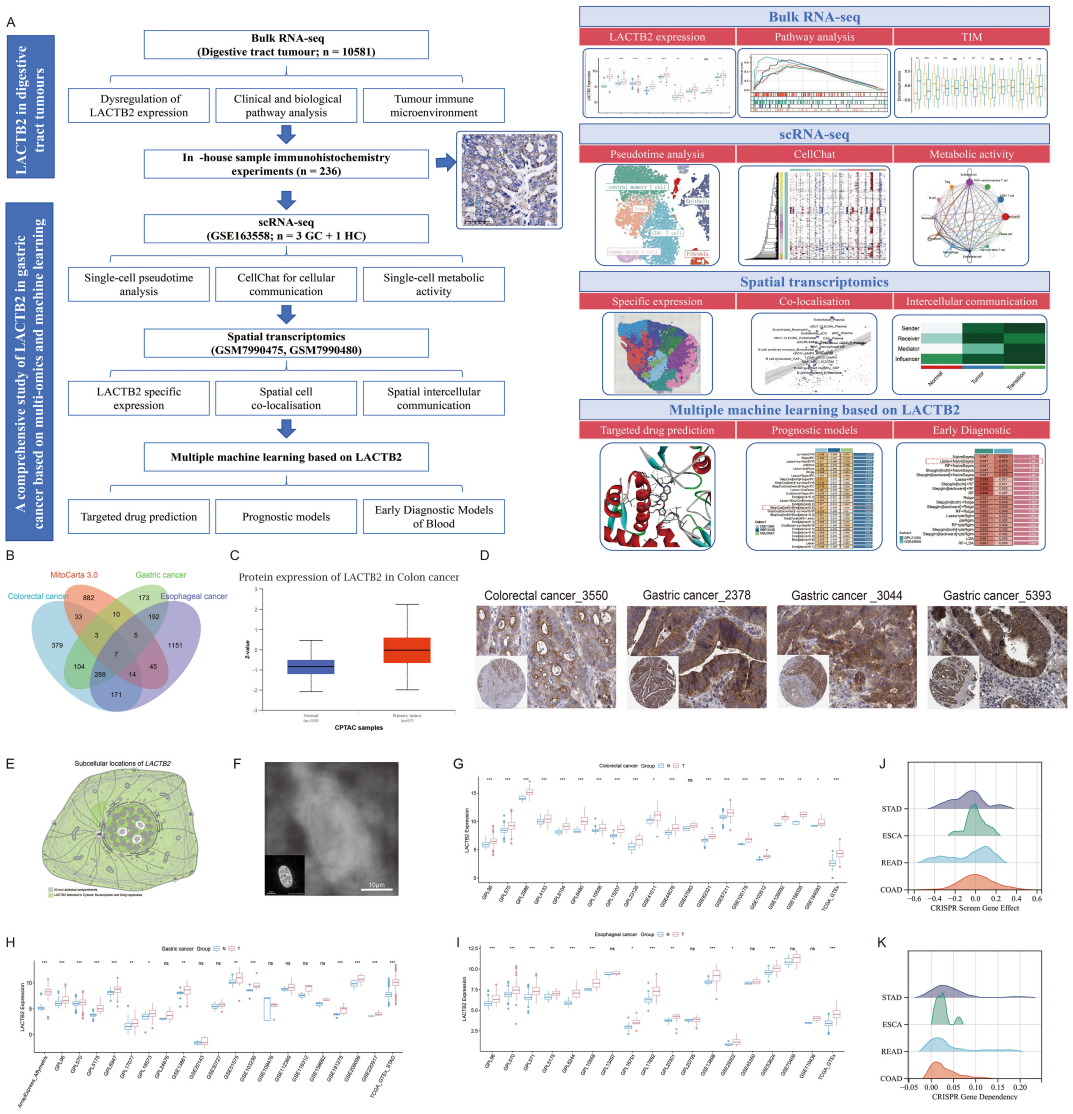
LACTB2 mRNA and protein are specifically expressed in digestive tract tumours. **(A)** Flow chart of this study. **(B)** The intersection of mitochondria-associated genes and highly expressed genes in digestive tract tumours. **(C)** Protein levels of LACTB2 in colon cancer. **(D)** Immunohistochemical staining of LACTB2 in colorectal and gastric cancers (GC). **(E)** Subcellular protein localisation. **(F)** Protein localisation prediction based on OpenGPS. **(G)** Differential expression of LACTB2 mRNA in colorectal cancer (CRC). **(H)** Differential expression of LACTB2 mRNA in GC. **(I)** Differential expression of LACTB2 mRNA in esophageal cancer (ESCA). **(J)** CRISPR Screen Gene Effect. **(K)** CRISPR Gene Dependency. ^ns/NS^p > 0.05, *p < 0.05, **p < 0.01, ***p < 0.001, ****p < 0.0001.

Based on transcriptomic profiles from TCGA, this study performed genome-wide differential expression analysis, ultimately identifying highly expressed genes in colorectal cancer (CRC), ESCA, and GC. By intersecting these genes with the MitoCarta 3.0 gene set and applying CytoHubba ranking, we identified seven mitochondrial-related genes highly expressed in digestive tract tumours (**Figure 1B**), with LACTB2 being one of the hub genes. Protein data from the UALCAN platform confirmed upregulated LACTB2 expression in COAD (**Figure 1C**). IHC staining revealed higher LACTB2 protein expression with intense staining in CRC and GC tissues (**Figure 1D**). Subcellular localization analysis demonstrated predominant LACTB2 distribution in the cytosol, with partial localization to the nucleoplasm and Golgi apparatus (**Figure 1E**). Protein localization predictions further supported the predominant cytoplasmic localization of LACTB2 (**Figure 1F**).

Subsequently, across 169 included transcriptomic datasets, we observed significant overexpression of LACTB2 mRNA across the majority of cohorts (**Figures 1G–I**). Given the potential influence of individual datasets on the overall results, we calculated a pooled SMD across all cohorts. The results showed LACTB2 mRNA was significantly overexpressed in CRC (SMD = 1.38, 95% CI [1.14; 1.63]; **Supplementary Figure S2A**), ESCA (SMD = 1.20, 95% CI [0.89; 1.51]; **Supplementary Figure S3A**), and GC (SMD = 1.04, 95% CI [0.72; 1.36]; **Supplementary Figure S4A**). Egger’s test indicated no significant publication bias in the included CRC (*p* = 0.102, **Supplementary Figure S2B**), ESCA (*p* = 0.342, **Supplementary Figure S3B**), or GC (*p* = 0.586, **Supplementary Figure S4B**) datasets. Furthermore, during model evaluation, significant heterogeneity was observed in both the GPL570 and TCGA cohorts for CRC (**Supplementary Figures S2C, D**), ESCA (**Supplementary Figures S3C, D**), and GC (**Supplementary Figures S4C, D**). Considering the impact of different platforms, we performed leave-one-out sensitivity analysis, identifying these two cohorts as the primary sources of heterogeneity. Based on the differential expression pattern of LACTB2 mRNA between digestive tract tumour samples and controls, this study constructed cohort-specific ROC curves to evaluate the diagnostic efficacy of LACTB2 mRNA. Cross-cohort analyses revealed that LACTB2 mRNA exhibited significant diagnostic value in CRC (**Supplementary Figure S2E**), ESCA (**Supplementary Figure S3E**), and GC (**Supplementary Figure S4E**). sROC curves further confirmed the robust discriminative ability of LACTB2 mRNA for digestive tract malignancies (CRC AUC = 0.92, **Supplementary Figure S2F**; ESCA AUC = 0.88, **Supplementary Figure S3F**; GC AUC = 0.89, **Supplementary Figure S4F**), suggesting pan-cancer diagnostic potential of LACTB2 mRNA across tumours. Additionally, **Figure 1J** shows that LACTB2 knockout significantly attenuated proliferation viability in COAD, ESCA, and STAD cell lines (CRISPR screen gene effect < 0), with high experimental consistency (**Figure 1K**). These findings indicate an oncogenic function of LACTB2 in COAD, ESCA, and STAD.

### 3.2 LACTB2 overexpression was associated with clinical metastasis and upregulation of oncogenic signalling pathways in digestive tract tumours

**Figure 2:**
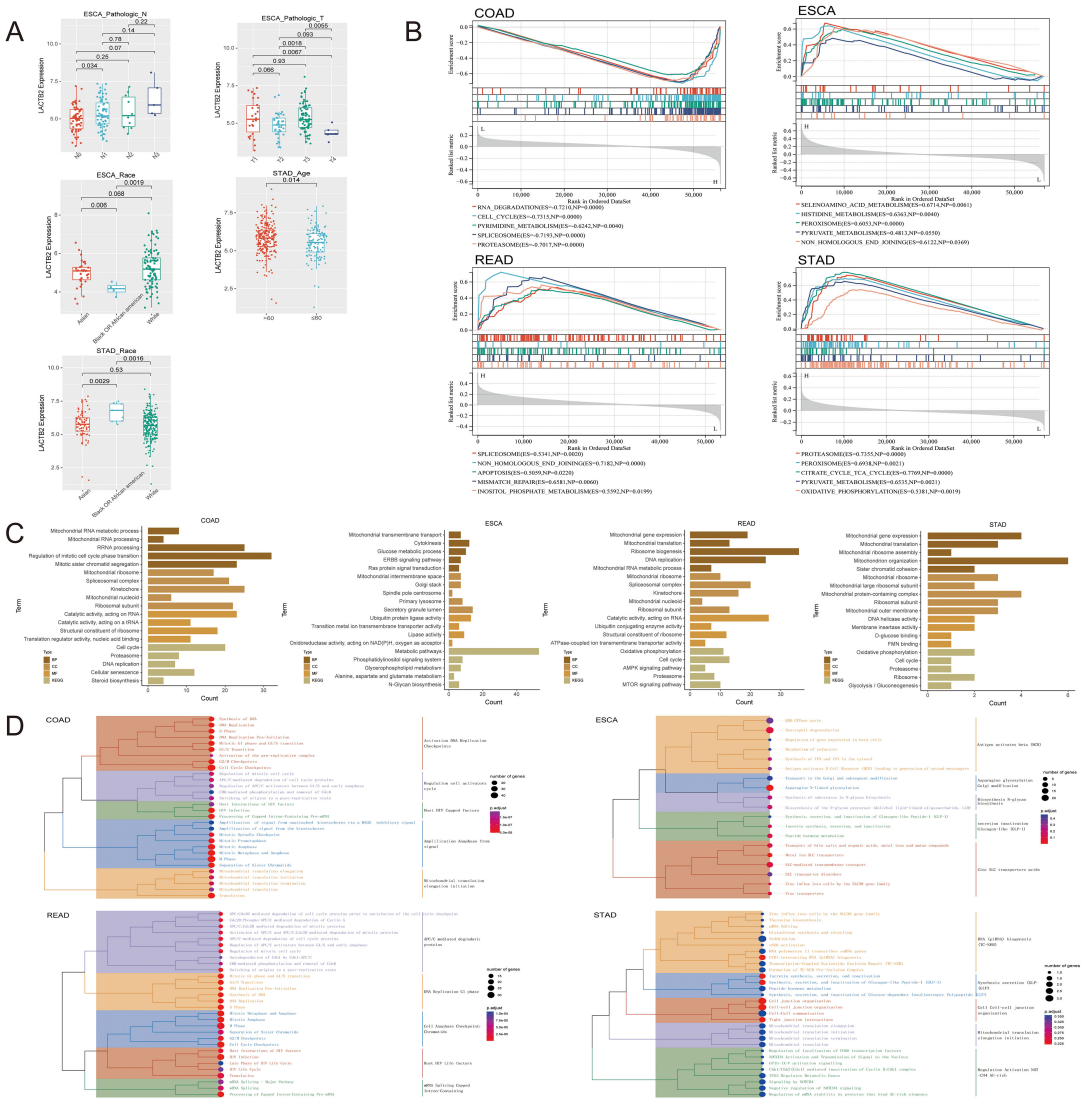
Clinical analysis and pathway enrichment of LACTB2 expression and highly expressed co-expression genes in multiple digestive tract tumours. **(A)** Scatter plot showing the relationship between LACTB2 expression and the clinical parameters of digestive tract tumours. **(B)** GSEA analysis. **(C)** GO and KEGG analysis. **(D)** Reactome enrichment analysis.

This study revealed associations between LACTB2 overexpression and specific clinicopathological features in digestive tract cancer patients. LACTB2 overexpression showed significant correlations with pathological characteristics in ESCA (**Table 2**) and STAD (**Table 3**), but not in COAD (**Table 4**) or READ (**Table 5**). In ESCA and STAD, elevated LACTB2 expression was significantly linked to early lymph node metastasis, tumour invasion depth, patient age, and ethnicity (**Figure 2A**). To investigate underlying molecular mechanisms, we performed enrichment analyses using multiple methodologies.

**Table 2:** Investigating the relationship between LACTB2 mRNA expression and clinical characteristics in patients with ESCA from the TCGA_ESCA_mRNA dataset.

Clinical features	LACTB2 mRNA epression			*t* (t-test) or *F *(ANOVA test)	*p* value
	Number	Mean	SD		
Age				1.431	0.1545
>60	77	5.336	1.048		
≤60	76	5.118	0.827		
Sex				-0.202	0.8401
Male	132	5.222	0.940		
Female	21	5.267	1.020		
BMI				0.729	0.4669
>30	23	5.334	0.979		
≤30	121	5.182	0.901		
Race				3.860	0.0235
White	97	5.249	0.956		
Asian	34	4.971	0.668		
Black or African American	4	4.153	0.334		
Clinical stage				1.371	0.2542
Stage I	16	5.218	1.091		
Stage II	67	5.083	0.946		
Stage III	53	5.432	0.940		
Stage IV	14	5.139	0.871		
history_of_esophageal_cancer				1.583	0.1161
Yes	11	5.510	0.970		
No	104	5.076	0.856		
Primary_pathology.lymph_node_metastasis_radiographic_evidence				-0.486	0.6280
Yes	47	5.172	0.895		
No	78	5.262	1.047		
Grade				0.921	0.4009
G1	15	4.936	0.697		
G2	62	5.229	0.909		
G3	42	5.313	1.014		
Primary_radiation_therapy				-1.698	0.0920
Yes	14	4.795	1.050		
No	109	5.269	0.975		
Pathologic_M				0.407	0.6843
M0	117	5.235	0.922		
M1	15	5.133	0.839		
Pathologic_N				4.074	0.0083
N0	62	5.009	0.851		
N1	66	5.352	0.959		
N2	10	5.460	1.121		
N3	5	6.339	1.216		
Pathologic_T				4.455	0.0050
T1	26	5.367	1.192		
T2	37	4.860	0.802		
T3	83	5.390	0.890		
T4	5	4.375	0.480		

**Table 3:** Investigating the relationship between LACTB2 mRNA expression and clinical characteristics in patients with STAD from the TCGA_STAD_mRNA dataset.

Clinical features	LACTB2 mRNA epression			*t* (t-test) or *F* (ANOVA test)	*p* value
	Number	Mean	SD		
Age				2.568	0.0107
>60	232	5.809	1.002		
≤60	111	5.509	1.034		
Sex				-0.527	0.5987
Male	219	5.697	1.048		
Female	128	5.757	0.968		
Grade				1.088	0.3381
G1	10	5.788	0.932		
G2	133	5.817	0.864		
G3	197	5.650	1.115		
Race				4.489	0.0120
White	216	5.726	0.973		
Asian	69	5.726	1.093		
Black or African American	9	6.641	0.660		
Clinical stage				0.182	0.9084
Stage I	52	5.702	0.999		
Stage II	99	5.646	0.872		
Stage III	137	5.745	1.107		
Stage IV	38	5.683	1.083		
Radiation_therapy				-0.198	0.8435
Yes	43	5.672	0.780		
No	163	5.706	1.044		
family_history_of_stomach_cancer				-0.063	0.9496
Yes	254	5.656	0.955		
No	14	5.673	0.898		
h_pylori_infection				0.201	0.8407
Yes	131	5.682	0.978		
No	18	5.634	0.814		
Primary_lymph_node_presentation_assessment				0.367	0.7138
Yes	309	5.705	1.033		
No	22	5.623	0.558		
Pathologic_M				0.7984	0.4252
M0	306	5.722	1.025		
M1	25	5.551	1.094		
Pathologic_N				0.905	0.4390
N0	104	5.672	0.926		
N1	90	5.588	0.986		
N2	68	5.841	1.054		
N3	68	5.758	1.129		
Pathologic_T				0.944	0.4195
T1	20	5.675	1.114		
T2	76	5.644	1.043		
T3	153	5.639	1.046		
T4	90	5.851	0.887		

**Table 4:** Investigating the relationship between LACTB2 mRNA expression and clinical characteristics in patients with COAD from the TCGA_COAD_mRNA dataset.

Clinical features	LACTB2 mRNA epression			*t* (t-test) or *F* (ANOVA test)	*p* value
	Number	Mean	SD		
Age				1.515	0.1306
>60	317	5.632	0.902		
≤60	137	5.493	0.899		
Sex				1.239	0.2162
Male	240	5.585	0.895		
Female	214	5.480	0.906		
BMI				0.761	0.4473
≥30	296	5.512	0.811		
<30	158	5.580	0.9747		
Race				1.416	0.2383
White	212	5,686	0.852		
Asian	11	5.754	0.925		
Black or African American	59	5.459	0.792		
American Indian or Alaska Native	1	4.948	/		
Clinical stage				1.491	0.2233
Stage I	75	5,649	0.889		
Stage II	176	5.502	0.866		
Stage III	128	5.546	0.902		
Stage IV	64	5.494	1.015		
Lymphatic_invasion				-1.22	0.2233
Yes	163	5.461	0.956		
No	248	5.571	0.853		
Primary_therapy_outcome_success				3.181	0.0431
CR	17	5.168	0.639		
PR	6	5.912	1.010		
PD	3	5.453	0.258		
SD	1	3.794	/		
Followup_treatment_success				2.23	0.0867
CR	119	5.496	0.911		
PD	32	5.761	0.776		
PR	11	5.278	1.095		
SD	3	4.543	0.979		
Primary_lymph_node_presentation_assessment				-1.351	0.1962
YES	430	5.527	0.909		
NO	15	5.797	0.753		
Pathologic_M				0.3545	0.7233
M0	206	3.118	1.014		
M1	11	3.006	1.068		
Pathologic_N				0.145	0.8647
N0	267	5.548	0.884		
N1	105	5.542	0.903		
N2	82	5.487	0.959		
Pathologic_T				1.393	0.2442
T1	11	5.377	0.808		
T2	77	5.719	0.990		
T3	309	5.466	0.859		
T4	56	2.944	1.004		

**Table 5:** Investigating the relationship between LACTB2 mRNA expression and clinical characteristics in patients with READ from the TCGA_READ_mRNA dataset.

Clinical features	LACTB2 mRNA epression			*t* (t-test) or *F* (ANOVA test)	*p* value
	Number	Mean	SD		
Age				-1.934	0.0548
>60	108	5.312	0.826		
≤60	58	5.576	0.858		
Sex				1.485	0.1395
Male	90	5.493	0.821		
Female	76	5.299	0.865		
BMI				0.406	0.6857
>30	15	5.711	0.685		
≤30	58	5.614	0.853		
Race				0.969	0.3837
White	81	5.576	0.770		
Asian	1	5.969	/		
Black or African American	6	6.008	0.894		
Clinical stage				1.095	0.3531
Stage I	30	5.183	0.958		
Stage II	51	5.405	0.702		
Stage III	51	5.457	0.947		
Stage IV	25	5.582	0.805		
History_of_colon_polyps				0.436	0.6636
Yes	32	5.450	0.932		
No	115	5.374	0.855		
lymphatic_invasion				-0.450	0.6535
Yes	65	5.363	0.876		
No	83	5.427	0.855		
venous_invasion				-0.939	0.3491
Yes	36	5.286	1.012		
No	108	5.444	0.820		
Primary_lymph_node_presentation_assessment				-1.736	0.0846
Yes	155	5.377	0.837		
No	4	6.120	1.189		
Pathologic_M				-1.025	0.3068
M0	126	5.351	0.868		
M1	24	5.548	0.828		
Pathologic_N				1.587	0.2078
N0	84	5.318	0.796		
N1	45	5.400	0.979		
N2	34	5.625	0.780		
Pathologic_T				2.092	0.1034
T1	9	5.383	0.795		
T2	28	5.054	0.993		
T3	114	5.496	0.789		
T4	14	5.401	0.917		

GSEA performed separately across the four cancer types revealed that LACTB2 overexpression activates genome stability regulation systems, proteostasis networks, and metabolic hub pathways in digestive tract tumours (**Figure 2B**). To enhance analytical depth and identify functional modules, we conducted KEGG, GO, and Reactome enrichment analyses on LACTB2-associated high-expression correlated genes (HECEGs) in each tumour type (maximum gene set = 500; **Figures 2C, D**). Critically, HECEG pathway enrichment demonstrated significant overlap across digestive tract tumours, converging on three core functional modules (regulation of cell cycle progression and genome stability, mitochondrial-nuclear gene expression coupling, metabolic-protein network regulation). The synergistic operation of these modules—cell cycle propels proliferation, mitochondrial-nuclear coupling sustains energy production, and metabolic-protein network confers adaptability—establishes an oncogenic circuit that propagates tumorigenesis across tumours.

Collectively, we propose that LACTB2 orchestrates aberrant cell cycle activation, mitochondria-nuclear translational coupling, and metabolic-proteostatic adaptation to assemble a pan-cancer malignant proliferation-survival circuitry, revealing an evolutionarily conserved oncogenic mechanism of LACTB2.

### 3.3 LACTB2 expression correlated with the TME in digestive tract tumours

**Figure 3:**
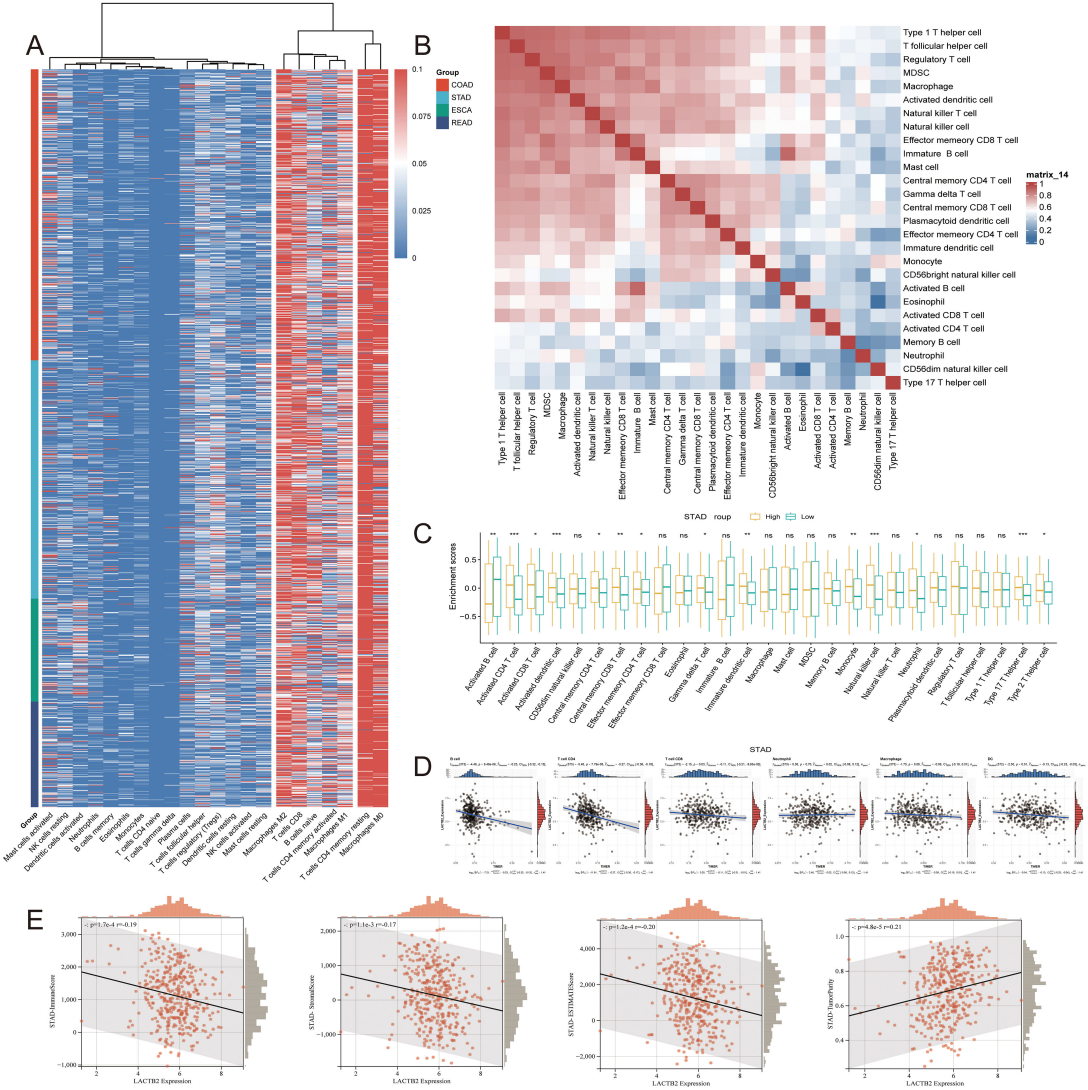
Immunocorrelation analysis of LACTB2 expression in digestive tract tumours. **(A)** Assessment of immune infiltration abundance in digestive tract tumours based on the “CIBERSORT” package. **(B)** Calculation of immune cell functional activity based on ssGSEA. **(C)** Differences in infiltration by different immune cells in digestive tract tumours between high- and low-LACTB2 expression groups. **(D)** TIMER analysis exploring the correlation of the relative infiltration of multiple immune cells. **(E)** STAD immune microenvironment analysis. ^ns/NS^p > 0.05, *p < 0.05, **p < 0.01, ***p < 0.001, ****p < 0.0001.

Given the pan-cancer influence of LACTB2, we quantified immune infiltration in digestive tract tumours by four distinct algorithms (CIBERSORT, EPIC, MCP-counter, and QUANTIseq). Immunological profiling revealed elevated absolute infiltration proportions of B cells naïve, macrophages, CD4/CD8 T cells, and neutrophils (**Figure 3A**; **Supplementary Figures S5A–C**), suggesting that the four digestive tract tumour types may exhibit an immune-hot tumours phenotype. Furthermore, we observed that the functional activity of multiple T cell types in STAD was positively correlated (**Figure 3B**). Compared to the low LACTB2 group, these T cell types exhibited significantly higher infiltration scores in the high LACTB2 group of STAD (**Figure 3C**). Identical findings were observed across the other three gastrointestinal cancers (**Supplementary Figure S5D**). TIMER analysis revealed that the relative infiltration of multiple immune cell types negatively correlated with LACTB2 expression in STAD (**Figure 3D**), whilst showing positive correlations in COAD, ESCA, and READ (**Supplementary Figure S5E**). Concurrently, LACTB2 expression negatively correlated with the overall immune score, mechanism score, and ESTIMATE score of the STAD TME, whilst significantly positively correlating with tumour purity (**Figure 3E**; **Supplementary Figure S5F**). The TIDE score was higher in the LACTB2 overexpression group (**Supplementary Figure S9C**). In GC, although certain T-cell functional scores are elevated, LACTB2 overexpression is associated with reduced overall immune infiltration and increased tumour purity. Based on these observations, we propose a research hypothesis that LACTB2 may promote immune escape in GC by shaping a microenvironment characterized by low immune infiltration and high tumour purity.

### 3.4 Validation of LACTB2 overexpression in In-House GC clinical specimens by IHC staining and exploration of upstream transcriptional regulation

**Figure 4:**
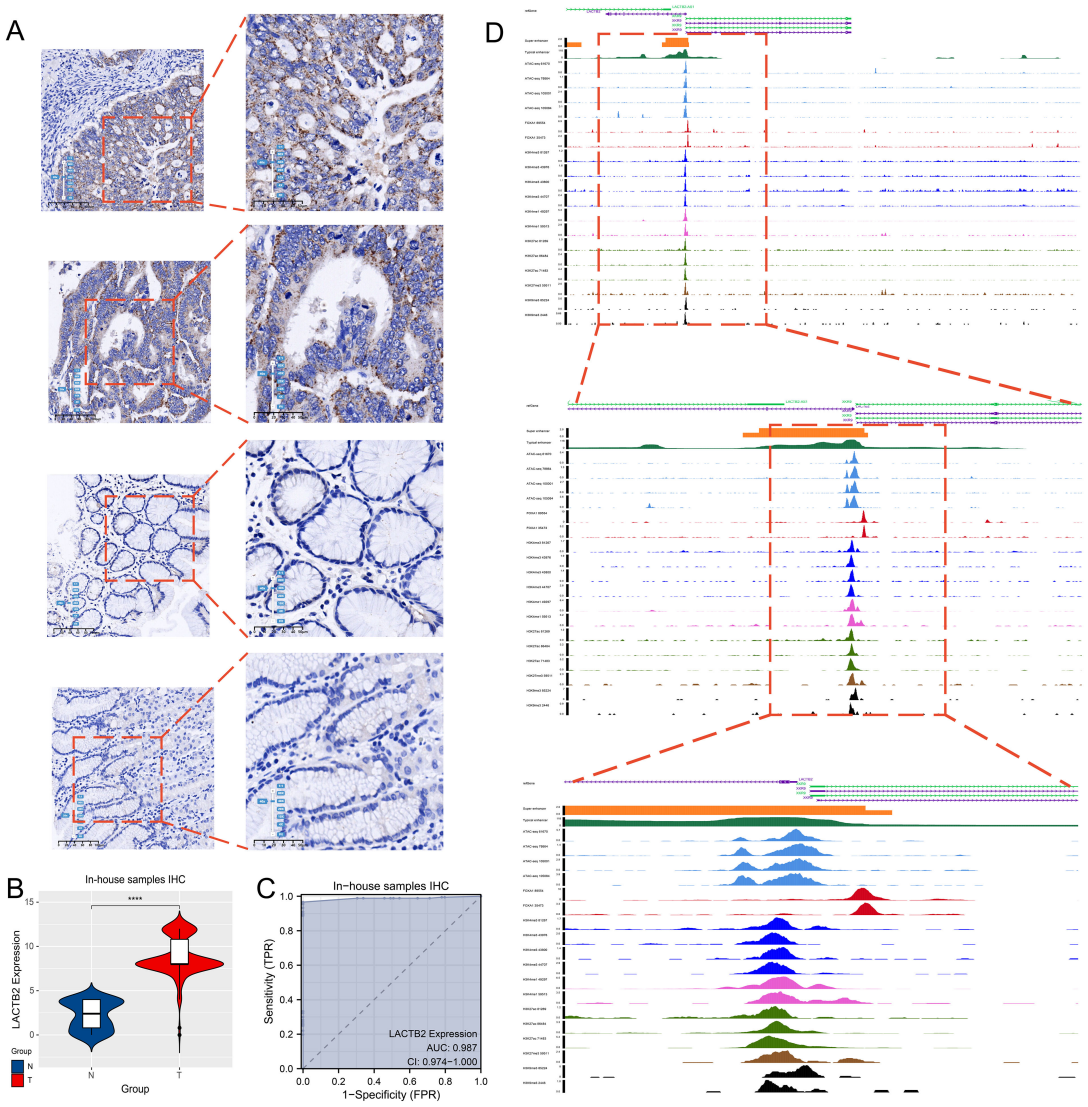
In-house immunohistochemistry (IHC) experiments were performed to verify the overexpression of LACTB2 and its upstream regulatory mechanism. **(A)** IHC staining of GC and control samples. **(B)** Protein expression levels of LACTB2 in GC and control samples. **(C)** ROC curves of in-house IHC. **(D)** Histone modifications were probed to investigate the potential transcriptional regulatory mechanism of LACTB2. ^ns/NS^p > 0.05, *p < 0.05, **p < 0.01, ***p < 0.001, ****p < 0.0001.

Given the critical role of LACTB2 in STAD, we validated its protein expression by 236 in-house GC clinical specimens. Microscopic analysis revealed weak positive staining in control tissues versus intensely strong positivity in tumour tissues (**Figure 4A**), with highly significant intergroup differences (p < 0.0001; **Figure 4B**). The ROC curve constructed from quantitative IHC scores confirmed exceptional diagnostic efficacy (AUC = 0.987, 95% CI [0.974–1.000]; **Figure 4C**). Primary data for all specimens were documented in **Table 6**.

**Table 6:** Raw data from internal immunohistochemical staining.

Sample ID	Group	Age	Gender	Pathology grade	TNM_T	TNM_N	TNM_M	LACTB2 protein levels
Tumour patient 1	GC	35	M	I	T2	N0	M0	8
Tumour patient 2	GC	36	M	I	T3	N0	M0	7.6
Tumour patient 3	GC	41	M	I	T2	N0	M0	8
Tumour patient 4	GC	45	M	I	T3	N1	M0	8
Tumour patient 5	GC	52	M	I	T2	N0	M0	12
Tumour patient 6	GC	54	M	I	T2	N1	M0	8
Tumour patient 7	GC	55	M	I	T3	N0	M0	8
Tumour patient 8	GC	55	M	I	T3	N0	M0	8
Tumour patient 9	GC	56	F	I	T2	N2	M0	8.8
Tumour patient 10	GC	57	M	I	T2	N1	M0	12
Tumour patient 11	GC	62	M	I	T1	N0	M0	4
Tumour patient 12	GC	64	M	I	T3	N1	M0	9.2
Tumour patient 13	GC	64	M	I	T3	N1	M0	12
Tumour patient 14	GC	66	M	I	T3	N1	M0	8
Tumour patient 15	GC	70	F	I	T2	N0	M0	8.4
Tumour patient 16	GC	81	M	I	T3	N1	M0	8
Tumour patient 17	GC	82	F	I	T1	N0	M0	11.6
Tumour patient 18	GC	36	M	I~II	T3	N0	M0	11.6
Tumour patient 19	GC	52	M	I~II	T2	N1	M0	8
Tumour patient 20	GC	55	F	I~II	T3	N0	M0	8
Tumour patient 21	GC	58	M	I~II	T2	N1	M0	8
Tumour patient 22	GC	63	M	I~II	T1	N0	M0	8
Tumour patient 23	GC	65	M	I~II	T3	N1	M0	8.8
Tumour patient 24	GC	65	M	I~II	T3	N2	M0	6.4
Tumour patient 25	GC	69	F	I~II	T3	N1	M0	8
Tumour patient 26	GC	70	M	I~II	T2	N0	M0	8
Tumour patient 27	GC	77	M	I~II	T3	N1	M0	8
Tumour patient 28	GC	81	M	I~II	T3	N1	M0	10
Tumour patient 29	GC	44	M	II	T3	N0	M0	12
Tumour patient 30	GC	44	M	II	T3	N2	M0	12
Tumour patient 31	GC	45	F	II	T3	N1	M0	12
Tumour patient 32	GC	47	M	II	T2	N2	M0	12
Tumour patient 33	GC	50	M	II	T3	N1	M0	0.8
Tumour patient 34	GC	50	F	II	T2	N0	M0	12
Tumour patient 35	GC	50	M	II	T3	N1	M0	12
Tumour patient 36	GC	51	M	II	T3	N1	M0	7.2
Tumour patient 37	GC	51	M	II	T2	N0	M0	12
Tumour patient 38	GC	52	M	II	T3	N1	M0	8
Tumour patient 39	GC	52	M	II	T3	N1	M0	8
Tumour patient 40	GC	54	M	II	T4	N1	M0	8
Tumour patient 41	GC	56	M	II	T2	N0	M0	12
Tumour patient 42	GC	56	F	II	T3	N1	M0	8
Tumour patient 43	GC	56	M	II	T2	N1	M0	12
Tumour patient 44	GC	56	M	II	T2	N0	M0	12
Tumour patient 45	GC	56	F	II	T2	N0	M0	12
Tumour patient 46	GC	58	M	II	T3	N0	M0	12
Tumour patient 47	GC	60	M	II	T3	N1	M0	8
Tumour patient 48	GC	60	M	II	T3	N1	M0	12
Tumour patient 49	GC	61	M	II	T2	N0	M0	8
Tumour patient 50	GC	61	M	II	T2	N1	M0	8
Tumour patient 51	GC	63	M	II	T3	N1	M0	12
Tumour patient 52	GC	65	M	II	T3	N1	M0	12
Tumour patient 53	GC	65	M	II	T3	N1	M0	8
Tumour patient 54	GC	66	M	II	T3	N1	M0	8
Tumour patient 55	GC	68	M	II	T3	N0	M0	11.6
Tumour patient 56	GC	68	M	II	T3	N1	M0	8
Tumour patient 57	GC	68	M	II	T2	N0	M0	12
Tumour patient 58	GC	69	M	II	T2	N0	M0	12
Tumour patient 59	GC	70	M	II	T2	N0	M0	12
Tumour patient 60	GC	70	M	II	T3	N2	M0	8
Tumour patient 61	GC	72	F	II	T2	N0	M0	10
Tumour patient 62	GC	72	F	II	T2	N0	M0	8
Tumour patient 63	GC	72	M	II	T2	N0	M0	8
Tumour patient 64	GC	73	F	II	T3	N1	M0	8
Tumour patient 65	GC	76	F	II	T3	N2	M0	10.8
Tumour patient 66	GC	43	M	II~III	T3	N0	M0	8
Tumour patient 67	GC	43	M	II~III	T3	N0	M0	8
Tumour patient 68	GC	47	F	II~III	T3	N0	M0	8
Tumour patient 69	GC	51	F	II~III	T3	N1	M0	8.4
Tumour patient 70	GC	52	F	II~III	T3	N0	M0	8
Tumour patient 71	GC	52	M	II~III	T3	N1	M0	8
Tumour patient 72	GC	53	M	II~III	T2	N0	M0	9.6
Tumour patient 73	GC	58	M	II~III	T3	N0	M0	8
Tumour patient 74	GC	60	M	II~III	T3	N2	M0	8
Tumour patient 75	GC	60	M	II~III	T2	N0	M0	8
Tumour patient 76	GC	61	M	II~III	T3	N1	M0	12
Tumour patient 77	GC	61	M	II~III	T3	N0	M0	8
Tumour patient 78	GC	62	M	II~III	T3	N0	M0	8
Tumour patient 79	GC	62	F	II~III	T2	N0	M0	12
Tumour patient 80	GC	65	M	II~III	T3	N1	M0	12
Tumour patient 81	GC	65	F	II~III	T3	N0	M0	4
Tumour patient 82	GC	67	F	II~III	T3	N1	M0	12
Tumour patient 83	GC	67	M	II~III	T3	N1	M0	8
Tumour patient 84	GC	76	M	II~III	T3	N0	M0	8
Tumour patient 85	GC	24	F	III	T3	N1	M0	8
Tumour patient 86	GC	30	F	III	T2	N0	M0	8
Tumour patient 87	GC	35	F	III	T3	N1	M0	8
Tumour patient 88	GC	37	M	III	T3	N1	M0	8
Tumour patient 89	GC	39	M	III	T2	N1	M0	11.2
Tumour patient 90	GC	40	M	III	T3	N0	M0	6.4
Tumour patient 91	GC	42	M	III	T3	N1	M0	8
Tumour patient 92	GC	42	F	III	T3	N2	M0	11.6
Tumour patient 93	GC	45	M	III	T3	N2	M0	12
Tumour patient 94	GC	45	F	III	T3	N0	M0	12
Tumour patient 95	GC	45	F	III	T3	N1	M0	12
Tumour patient 96	GC	45	M	III	T2	N0	M0	8
Tumour patient 97	GC	47	M	III	T3	N1	M0	8
Tumour patient 98	GC	48	M	III	T3	N1	M0	10.4
Tumour patient 99	GC	48	M	III	T3	N1	M0	12
Tumour patient 100	GC	48	M	III	T3	N1	M0	11.2
Tumour patient 101	GC	48	M	III	T3	N1	M0	8
Tumour patient 102	GC	49	M	III	T3	N1	M0	6
Tumour patient 103	GC	50	M	III	T3	N1	M0	8
Tumour patient 104	GC	50	F	III	T3	N1	M0	8
Tumour patient 105	GC	51	F	III	T3	N1	M0	4
Tumour patient 106	GC	51	F	III	T3	N1	M0	8
Tumour patient 107	GC	52	M	III	T2	N0	M0	8
Tumour patient 108	GC	52	M	III	T3	N1	M0	8
Tumour patient 109	GC	53	F	III	T3	N1	M0	8
Tumour patient 110	GC	54	M	III	T3	N1	M0	8
Tumour patient 111	GC	54	M	III	T3	N1	M0	8
Tumour patient 112	GC	54	M	III	T3	N1	M0	8
Tumour patient 113	GC	55	M	III	T3	N1	M0	6
Tumour patient 114	GC	55	F	III	T3	N1	M0	8
Tumour patient 115	GC	56	M	III	T3	N1	M0	12
Tumour patient 116	GC	58	M	III	T2	N2	M0	8
Tumour patient 117	GC	59	F	III	T3	N2	M0	8
Tumour patient 118	GC	60	M	III	T2	N0	M0	8.8
Tumour patient 119	GC	60	M	III	T3	N2	M0	8
Tumour patient 120	GC	60	M	III	T3	N2	M0	12
Tumour patient 121	GC	60	M	III	T2	N1	M0	8
Tumour patient 122	GC	61	M	III	T2	N0	M0	8
Tumour patient 123	GC	61	M	III	T3	N0	M0	8
Tumour patient 124	GC	62	M	III	T3	N0	M0	9.2
Tumour patient 125	GC	62	M	III	T3	N1	M0	5.6
Tumour patient 126	GC	62	M	III	T2	N2	M0	8
Tumour patient 127	GC	63	M	III	T2	N0	M0	6
Tumour patient 128	GC	63	M	III	T3	N1	M0	6.8
Tumour patient 129	GC	64	M	III	T3	N1	M0	12
Tumour patient 130	GC	68	M	III	T3	N2	M0	8
Tumour patient 131	GC	68	M	III	T3	N0	M0	12
Tumour patient 132	GC	68	F	III	T3	N0	M0	11.2
Tumour patient 133	GC	70	M	III	T3	N2	M0	12
Tumour patient 134	GC	70	M	III	T2	N1	M0	8
Tumour patient 135	GC	70	F	III	T3	N0	M0	8
Tumour patient 136	GC	71	F	III	T3	N1	M0	8
Tumour patient 137	GC	71	M	III	T3	N0	M0	8
Tumour patient 138	GC	71	F	III	T3	N1	M0	8
Tumour patient 139	GC	72	F	III	T2	N0	M0	8
Tumour patient 140	GC	72	M	III	T3	N2	M0	8
Tumour patient 141	GC	73	M	III	T2	N1	M0	6.4
Tumour patient 142	GC	74	M	III	T3	N1	M0	12
Tumour patient 143	GC	75	M	III	T3	N1	M0	6.8
Tumour patient 144	GC	77	M	III	T3	N1	M0	4.4
Tumour patient 145	GC	78	M	III	T3	N1	M0	8
Tumour patient 146	GC	78	M	III	T3	N2	M0	8
Tumour patient 147	GC	83	M	III	T2	N0	M0	8
Tumour patient 148	GC	65	M	III~IV	T3	N1	M0	8
Tumour patient 149	GC	29	M		T3	N1	M0	11.2
Tumour patient 150	GC	42	F		T3	N2	M0	6
Tumour patient 151	GC	45	M		T3	N0	M0	8
Tumour patient 152	GC	60	M		T2	N1	M0	9.6
Tumour patient 153	GC	53	F		T2	N0	M0	8
Tumour patient 154	GC	46	M		T3	N0	M0	8
Tumour patient 155	GC	50	M		T2	N0	M0	8
Tumour patient 156	GC	51	M		T3	N1	M0	8
Tumour patient 157	GC	59	M		T3	N0	M0	8
Tumour patient 158	GC	61	M		T2	N0	M0	8
Tumour patient 159	GC	65	F		T3	N1	M0	4
Tumour patient 160	GC	69	F		T3	N2	M0	8
Tumour patient 161	GC	70	F		T3	N1	M0	9.2
Tumour patient 162	GC	70	F		T2	N1	M0	8
Tumour patient 163	GC	44	F		T2	N1	M0	8
Tumour patient 164	GC	40	F		T3	N1	M0	8
Tumour patient 165	GC	50	M		T3	N1	M0	8
Tumour patient 166	GC	56	M		T2	N0	M0	8
Tumour patient 167	GC	54	M		T3	N1	M0	8
Tumour patient 168	GC	64	F		T3	N1	M0	8
Tumour patient 169	GC	75	M		T3	N1	M0	6.4
Tumour patient 170	GC	48	F		T2	N0	M0	8
Tumour patient 171	GC	35	M		T3	N1	M0	8
Tumour patient 172	GC	62	M		T3	N2	M0	8
Tumour patient 173	GC	43	M		T2	N0	M0	8
Tumour patient 174	GC	65	M		T3	N2	M0	0
Tumour patient 175	GC							8
Tumour patient 176	GC							8.8
Tumour patient 177	GC							11.2
Control patient 1	Control	6	M					1.6
Control patient 2	Control	63	M					1.6
Control patient 3	Control	38	M					0
Control patient 4	Control	67	M					0.8
Control patient 5	Control	53	M					1.2
Control patient 6	Control	60	F					0.8
Control patient 7	Control	45	M					1.2
Control patient 8	Control	38	M					0
Control patient 9	Control	22	M					0
Control patient 10	Control							0
Control patient 11	Control							1.6
Control patient 12	Control							0.4
Control patient 13	Control							3.6
Control patient 14	Control							3.2
Control patient 15	Control							2.4
Control patient 16	Control							1.6
Control patient 17	Control							4
Control patient 18	Control							4
Control patient 19	Control							4
Control patient 20	Control							4
Control patient 21	Control							1.2
Control patient 22	Control							4
Control patient 23	Control							0
Control patient 24	Control							3.2
Control patient 25	Control							0
Control patient 26	Control							0
Control patient 27	Control							4
Control patient 28	Control							1.2
Control patient 29	Control							4
Control patient 30	Control							4
Control patient 31	Control							4
Control patient 32	Control							4
Control patient 33	Control							4
Control patient 34	Control							4
Control patient 35	Control							4
Control patient 36	Control							3.2
Control patient 37	Control							4
Control patient 38	Control							0.8
Control patient 39	Control							0
Control patient 40	Control							0
Control patient 41	Control							0
Control patient 42	Control							3.2
Control patient 43	Control							0
Control patient 44	Control							1.6
Control patient 45	Control							4
Control patient 46	Control							4
Control patient 47	Control							4
Control patient 48	Control							3.2
Control patient 49	Control							0
Control patient 50	Control							4
Control patient 51	Control							0.8
Control patient 52	Control	51	M					1.6
Control patient 53	Control	74	M					2
Control patient 54	Control	49	F					3.6
Control patient 55	Control	76	M					2.8
Control patient 56	Control	70	M					2.8
Control patient 57	Control							2
Control patient 58	Control							3.2
Control patient 59	Control							3.2

Furthermore, intersecting LACTB2-predicted transcription factors (regulatory potential >0.8) from the Cistrome DB with GC highly expressed genes identified FOXA1 as the key regulator. In both GC samples and control samples, FOXA1 and LACTB2 expression exhibited a significant and consistent positive correlation (**Supplementary Figure S9D**). **Figure 4D** demonstrates distinct bimodal ATAC-seq peaks upstream of the LACTB2 TSS, indicating accessible chromatin regions. FOXA1 ChIP-seq exhibited a prominent unimodal peak at the TSS, confirming its direct binding and transcriptional control of the LACTB2 promoter. Moreover, integrative mapping of histone modifications, which included H3K4me3 (promoter mark), H3K4me1/H3K27ac (enhancer signatures), and H3K27me3/H3K9me3 (repressive marks), revealed a spatially organized co-localization of antagonistic regulatory elements at the upstream regulatory region of LACTB2, featuring diverse activating and repressive chromatin domains. At the same time, multi-omics integration via Chromloops revealed distinct typical enhancer and super enhancer anchored at the upstream of the LACTB2 TSS. Collectively, our IHC validation confirms LACTB2 overexpression in GC tissues, while epigenomic dissection reveals a multilayered regulatory architecture characterized by enhancer-promoter cooperativity and competitive repressive signaling.

### 3.5 LACTB2 overexpression drives malignant transformation of GC epithelium via an oncogenic-metabolic signalling network

**Figure 5:**
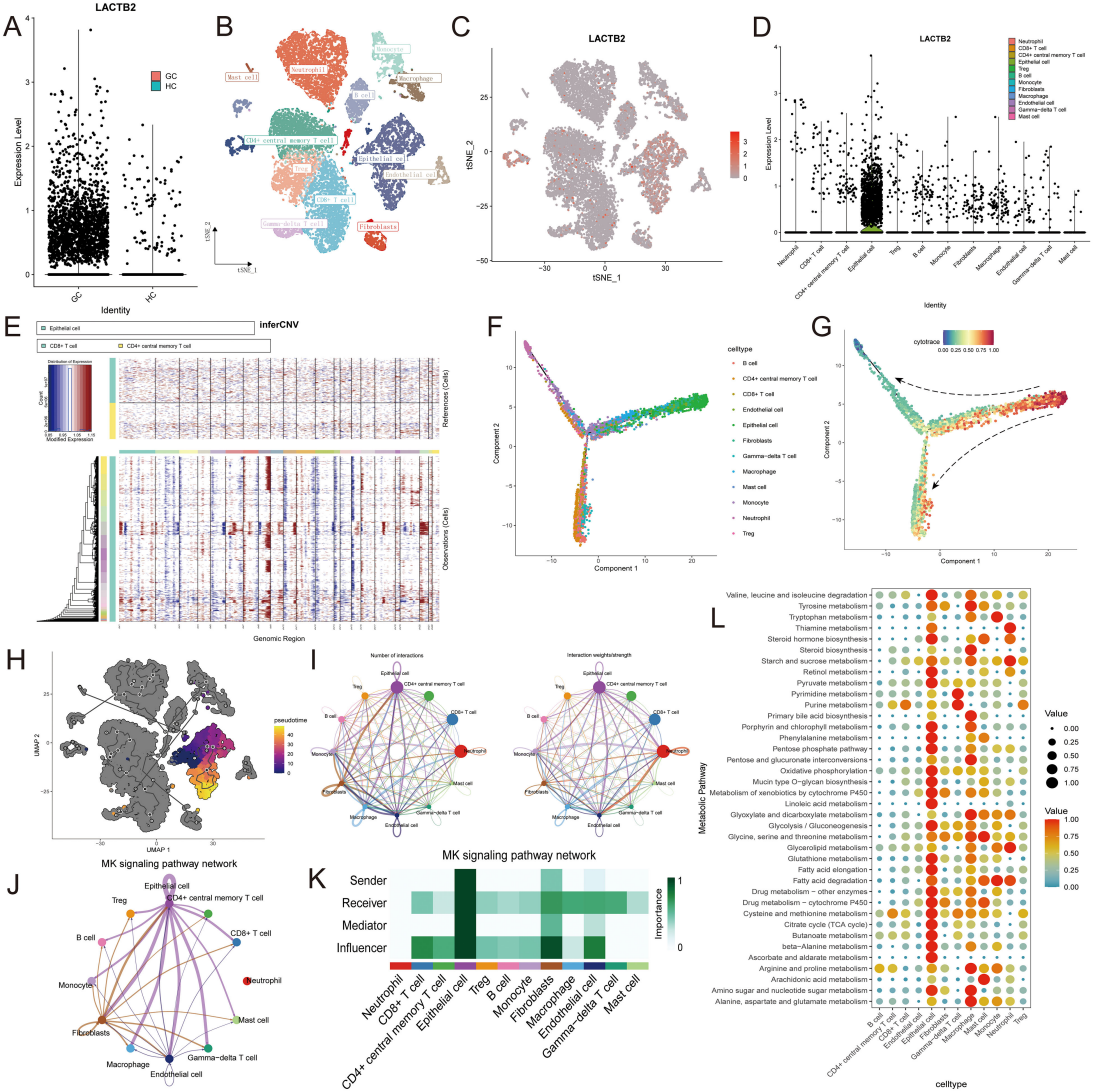
A comprehensive analysis of LACTB2 expression in GC based on scRNA-seq. **(A)** Violin plots of LACTB2 expression in GC and healthy control (HC) samples. **(B)** The incorporated cells were divided into 12 cell clusters. **(C)** UMAP plot of LACTB2 expression in cells. **(D)** Violin plots of LACTB2 expression in different cells. **(E)** InferCNV analysis to identify malignant tumours. **(F)** Differentiation trajectories of different cell clusters. **(G)** Proposed temporal sequence analysis of cell differentiation trajectories. **(H)** Mimetic time-series analysis targeting epithelial cell clusters. **(I)** Interaction based on communication between different cell clusters. **(J)** Hierarchical diagram of different cells involved in the MK signalling pathway. **(K)** Signalling roles of different cells in the MK signalling pathway. **(L)** Single-cell metabolic analysis.

Compared to normal samples, LACTB2 was significantly overexpressed in GC scRNA-seq data (**Figure 5A**). We identified 12 transcriptomically distinct cell clusters, with LACTB2 predominantly localized to epithelial cells (**Figures 5B–D**). These epithelial cells exhibited significant CNV—including recurrent amplifications and deletions overlapping known GC hotspots—indicating high malignant potential (**Figure 5E**). Using “CytoTRACE” to determine the differentiation origin of cell clusters, we found that LACTB2 was highly expressed in epithelial cell clusters with high differentiation potential and shows a declining expression trend (**Figures 5F, G**; **Supplementary Figures S6A–C**). Directed pseudotime trajectory analysis of epithelial clusters revealed a differentiation continuum progressing from early-stage (purple) to late-stage (yellow) phenotypes (**Figure 5H**). CellChat integration of ligand-receptor (L-R) pairs delineated multi-tiered cellular communication networks across GC clusters (**Figure 5I**). Epithelial cells, mainly via diverse L-R pairs like MDK-NCL, primarily participate in the MK signalling pathway (**Figure 5J**; **Supplementary Figure S6D**). In this pathway, epithelial cells play a quadruple role driving tumour progression. Epithelial cells sustain oncogenic signalling through autocrine loops and coordinate multicellular interactions (**Figure 5K**). Knockdown of LACTB2 significantly suppressed the MK signalling pathway in epithelial cells and reduced the expression of key genes (**Supplementary Figures S10A-C**). Furthermore, epithelial cells exhibited broad engagement in metabolic reprogramming pathways (**Figure 5L**). Collectively, LACTB2 specifically localized to malignant epithelial progenitors with high differentiation competence, and may drive transformation through MK pathway and metabolic dysregulation.

### 3.6 Spatial transcriptomics revealed LACTB2 expression, cell co-localization, and intercellular communication in the GC TME

**Figure 6:**
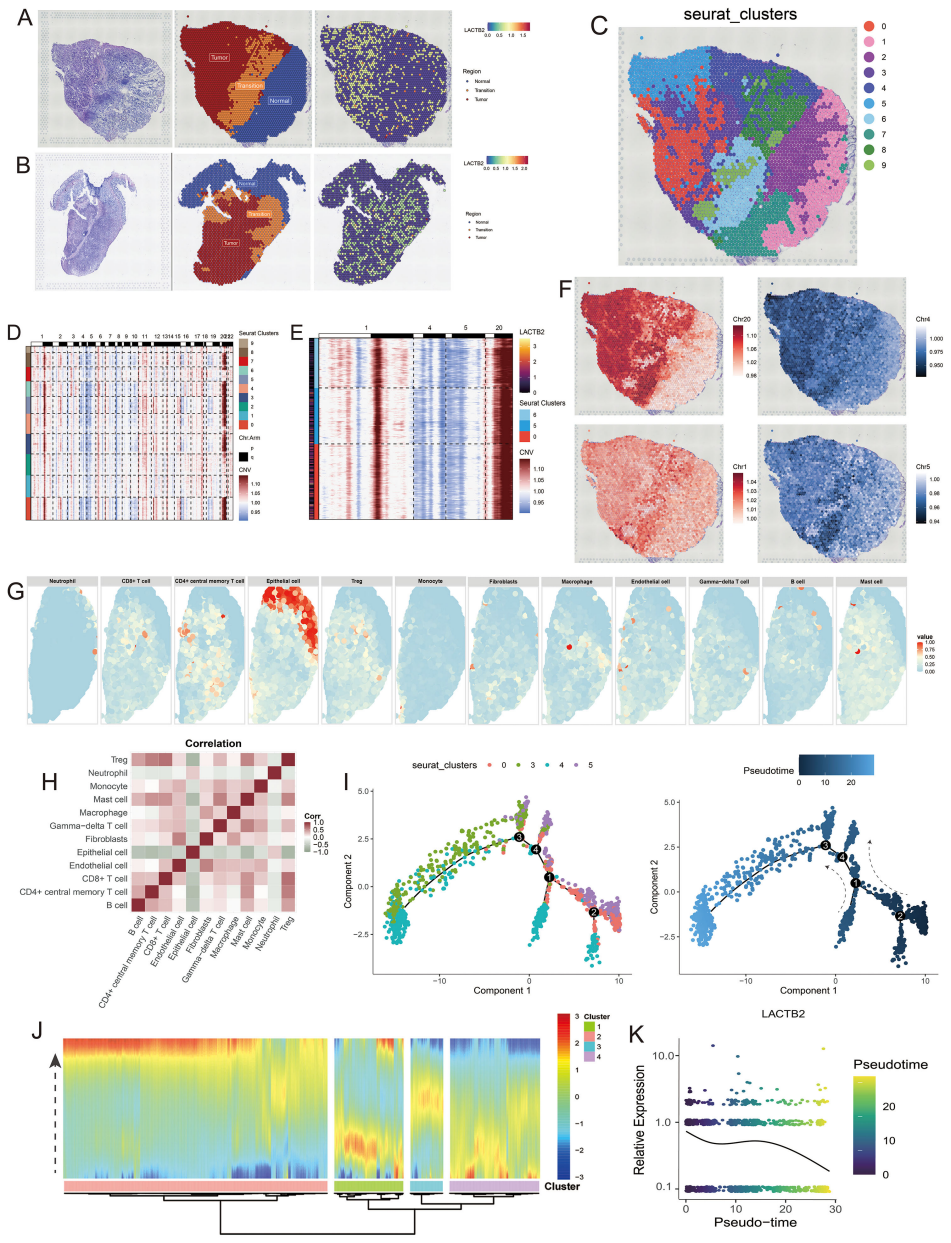
A comprehensive analysis of LACTB2 expression in GC based on spatial transcriptomics. **(A, B)** Spatial distribution of LACTB2 expression in GC tissues. **(C)** Incorporated cells were divided into ten cell clusters. **(D)** Copy number variation analysis (CNV) based on the ‘SPATA2’ package. **(E)** LACTB2 expression and CNV in different cell clusters. **(F)** Degree of copy variation in different regions of a single chromosome. **(G)** Distribution of different cells in the spatial transcriptome. **(H)** Cellular correlation analysis. **(I)** Proposed spatial time series analysis. The dark colour in the figure shows the starting point of the cell differentiation trajectory. **(J)** Heatmap showing four genomes functioning at different times in the tumour. The colour scale from blue to red indicates the relative expression level from low to high. **(K)** Gene expression trend of LACTB2 along a pseudo-time course.

Spatial transcriptomics further validated LACTB2 enrichment in tumour and transition zones (**Figures 6A, B**), consistent with scRNA-seq findings. Among the 10 identified cell clusters, clusters 0, 3, 5, 6, and 8 showed high malignancy and significant CNV fluctuations, with LACTB2 predominantly localised in tumour-area clusters 0 and 5 (**Figures 6C–F**). Meanwhile, the tumour and transition zones had substantial immune cell infiltration, with infiltration levels positively correlated (**Figures 6G, H**). Spatial pseudotime analysis revealed clusters 0 and 5 as differentiation origins, with LACTB2 expression decreasing along the pseudotime trajectory (**Figures 6I–K**).

**Figure 7:**
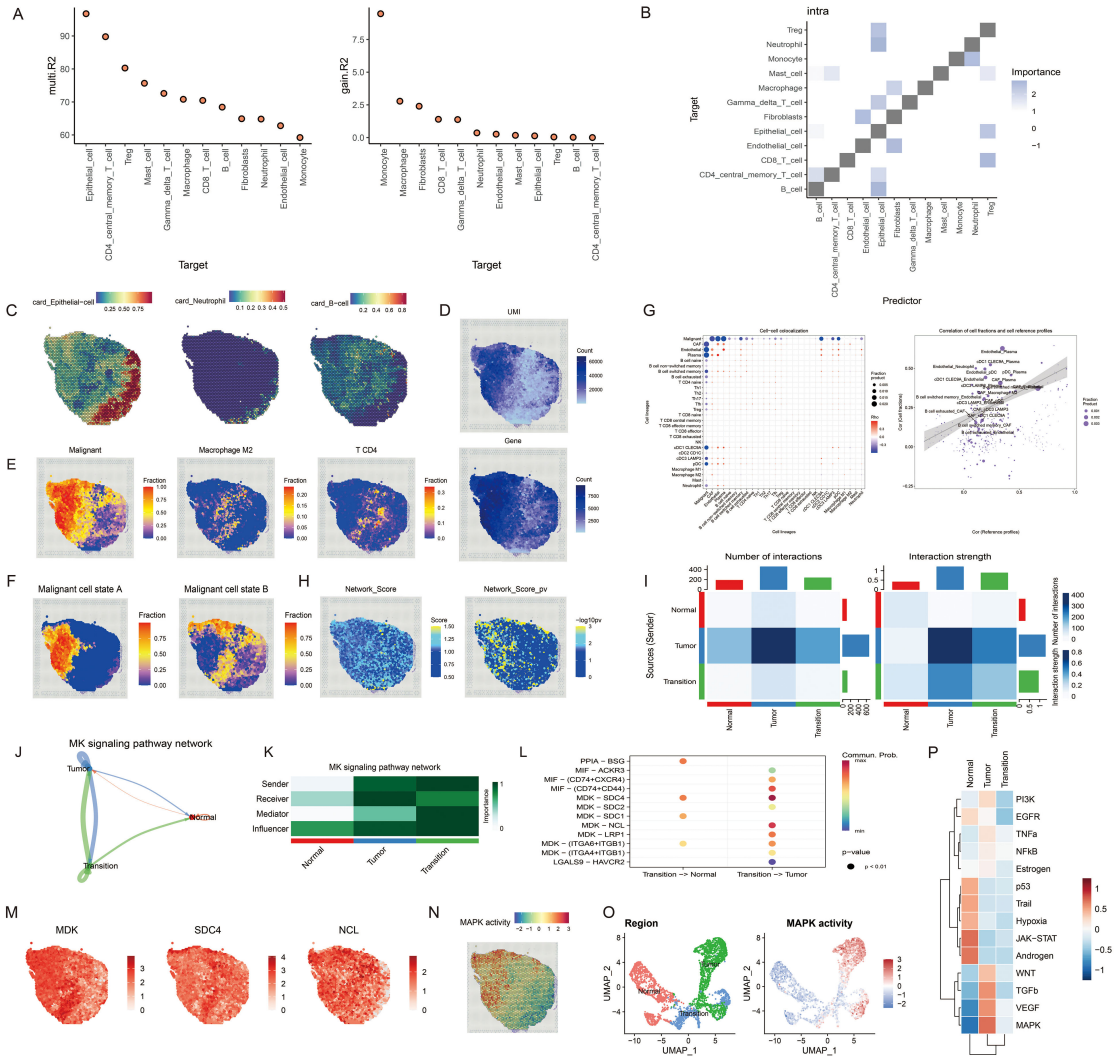
The spatial transcriptome was examined to explore the ability of GC cells to co-localise and communicate via spatial signals. **(A)** Single- and multi-view models explaining cellular colocalisation. **(B)** Prediction of cellular co-localisation targets in an internal view. **(C)** Spatial observation of epithelial cells, neutrophils and B cells based on the para-view algorithm. **(D)** Key quality control indicators. **(E)** Heatmap showing infiltration levels of macrophages, CD8+ T cells and CD4+ T cells in different distinctions. **(F)** Deconvolution to explore malignant cell status. **(G)** Finding co-localised cell type pairs based on the ‘SpaCET’ tool. **(H)** Analysis of L-R network enrichment within spatial transcriptome sites. **(I)** Number and intensity of interactions in different regions of space. **(J)** Association of the MK signalling pathway between different regions. **(K)** The signalling roles of different regions in the MK signalling pathway. **(L)** L-R pair bubble diagram. **(M)** Distribution of key gene expression. **(N)** Distribution of MAPK pathway activity. **(O)** UMAP plot showing the intensity of MAPK pathway activity in different regions. **(P)** A heatmap showing the activity of multiple signalling pathways in different regions.

To investigate cell co-localisation, we compared multi-view and single-view models. The multi-view model better explained epithelial cell variance, and monocyte localisation was more dependent on additional views (**Figure 7A**). Epithelial cells were reliable predictors for neutrophils (Importance = 2.78) and B cells (Importance = 2.74) (**Figure 7B**), and ParaView validation confirmed that areas with high epithelial proportions exhibited low neutrophil and B cell densities (**Figure 7C**). Malignant cell distribution aligned with defined tumour regions, with macrophages, CD8+ T cells, and CD4+ T cells showing significant infiltration in the tumour area (**Figures 7D, E**; **Supplementary Figure S7B**), consistent with immune analysis. Spatial pseudotemporal analysis further linked state A to early tumour stages and state B to late stages (**Figure 7F**). The tumour region harbours a rich network of L-R interactions, and the “SpaCET” tool identified endothelial cells and plasma cells as a highly co-localised pair (**Figures 7G, H**). Spatial permutation analysis revealed that immune cells were significantly excluded from LACTB2−high tumour core regions (observed mean distance = 9.30 vs. permuted mean = 7.48, p < 0.001; **Supplementary Figure S10D**).

In spatial cell communication, tumour and transition zones exhibited marked signalling exchanges, predominantly involving the MK signalling pathway and playing a multidimensional role, consistent with scRNA-seq analysis (**Figures 7I–K**). These zones participated in MK signalling via multiple ligand-receptor pairs, with key genes showing uniformly high expression (**Figures 7L, M**; **Supplementary Figures S7C, D**). Pathway activity analysis further revealed significantly elevated MAPK pathway activity specifically within the tumour region (**Figures 7N–P**). Taken together, spatial transcriptomics validated the scRNA-seq and immune infiltration findings while providing an integrative analysis of cellular co-localisation and the MK-MAPK signalling axis within the GC TME.

### 3.7 Dysregulation of LACTB2 expression adversely impacted prognosis in GC

**Figure 8:**
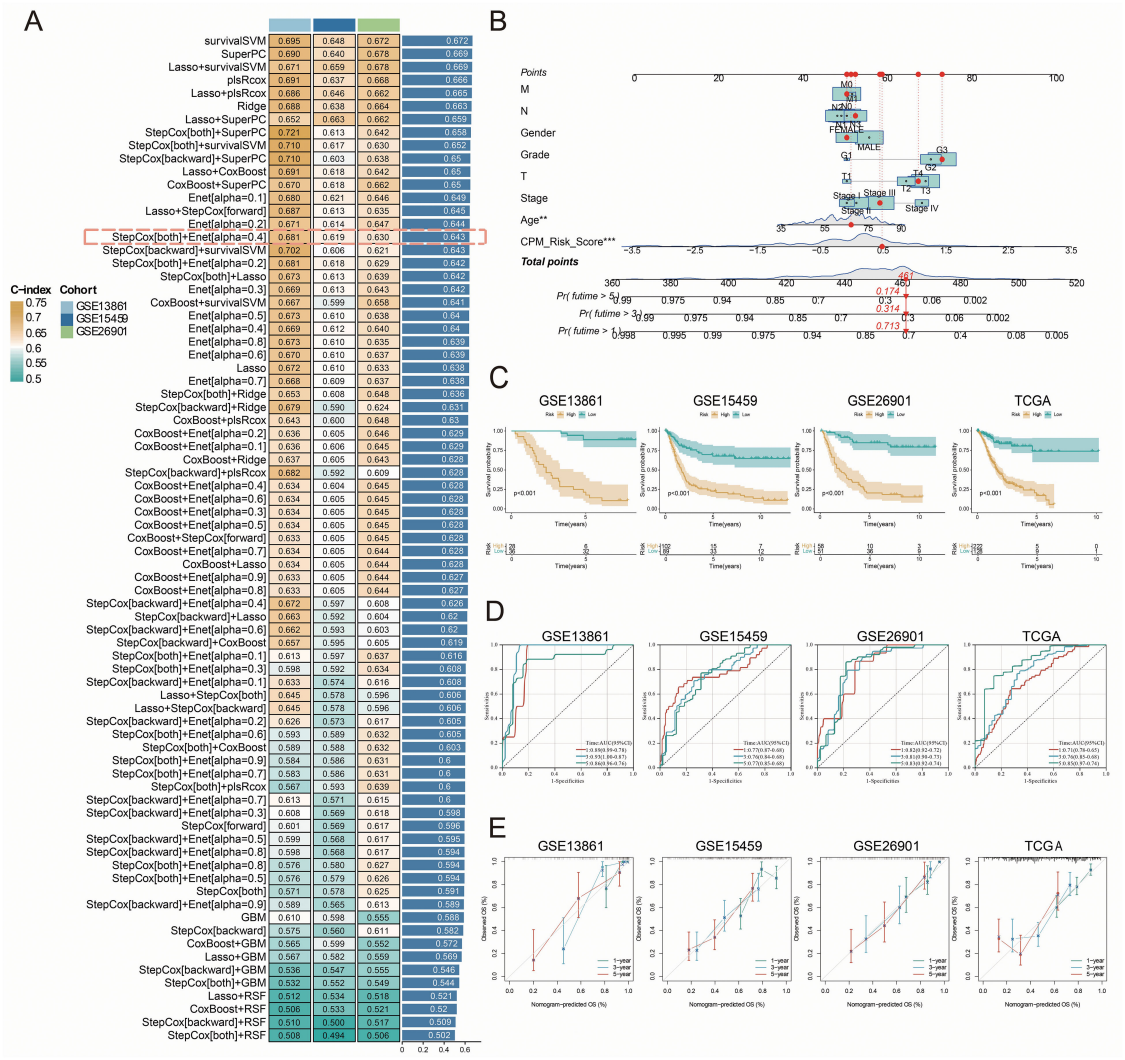
Analysis of LACTB2 in GC prognosis. **(A)** Construction of clinical prediction models based on 101 machine learning combinations. **(B)** Column line graph of the predictive model. **(C)** Survival curve analysis. **(D)** Time-dependent ROC curve analysis. **(E)** Correction curves.

To further elucidate the clinical significance of LACTB2, we implemented a leave-one-out cross-validation (LOOCV) framework to fit 101 machine learning predictive models. These models were applied to genes at the intersection of poor prognosis markers and highly expressed candidate genes (HECEGs) in GC for prognostic analysis (**Figure 8A**, **Table 7**). We selected a clinical prediction model (CPM) optimized for fewer candidate genes while maintaining superior predictive performance. The CPM “StepCox[both] + Enet[alpha=0.4]” incorporated 13 genes (BST1, CAST, CLDN6, GABARAPL2, GRB14, LACTB2, NETO2, NR1D1, NT5E, RHOBTB3, SEMA6A, SERPINC1, UPP1). A CPM risk score was subsequently calculated based on the expression levels of these genes.

**Table 7:** The performance of 101 predictive models in training and testing cohorts.

Model	GSE13861	GSE15459	GSE26901
Lasso+StepCox[both]	0.64452297	0.57822995	0.5958231
survivalSVM	0.69469965	0.64766891	0.67248157
CoxBoost+survivalSVM	0.66713781	0.59867641	0.65798526
Ridge	0.68833922	0.63789016	0.66412776
Lasso+survivalSVM	0.67137809	0.6588305	0.67764128
SuperPC	0.69045936	0.63986567	0.67764128
CoxBoost+Ridge	0.63745583	0.60450415	0.64348894
Enet[alpha=0.1]	0.67985866	0.62139471	0.64643735
CoxBoost+Enet[alpha=0.1]	0.6360424	0.60568945	0.64471744
Enet[alpha=0.2]	0.67137809	0.61438167	0.64692875
Enet[alpha=0.3]	0.66925795	0.61290004	0.64299754
CoxBoost+Enet[alpha=0.3]	0.63392226	0.60450415	0.64520885
CoxBoost+Enet[alpha=0.2]	0.6360424	0.6047017	0.64619165
Enet[alpha=0.4]	0.66925795	0.61151719	0.63980344
CoxBoost+Enet[alpha=0.4]	0.63392226	0.6043066	0.64545455
Lasso+CoxBoost	0.69116608	0.61803635	0.64176904
Enet[alpha=0.5]	0.67349823	0.60973923	0.63808354
CoxBoost+Enet[alpha=0.5]	0.63392226	0.60450415	0.64496314
Enet[alpha=0.6]	0.66996466	0.61013433	0.63685504
CoxBoost+Enet[alpha=0.6]	0.63392226	0.60499802	0.64471744
CoxBoost+Enet[alpha=0.7]	0.63392226	0.60489925	0.64422604
CoxBoost+Enet[alpha=0.8]	0.63321555	0.60489925	0.64422604
Enet[alpha=0.8]	0.67279152	0.60954168	0.63538084
Lasso	0.67208481	0.61003556	0.63267813
Enet[alpha=0.7]	0.66784452	0.60944291	0.63685504
CoxBoost+Enet[alpha=0.9]	0.63321555	0.60480047	0.64447174
CoxBoost+Lasso	0.63392226	0.60499802	0.64373464
Lasso+plsRcox	0.68621908	0.64598973	0.66240786
CoxBoost+plsRcox	0.64310954	0.59976294	0.64766585
CoxBoost+StepCox[forward]	0.63321555	0.60519557	0.64496314
Lasso+StepCox[forward]	0.6869258	0.61329514	0.63464373
CoxBoost+SuperPC	0.66996466	0.61843145	0.66167076
StepCox[forward]	0.60141343	0.56943896	0.61695332
plsRcox	0.69116608	0.63729751	0.66805897
Lasso+SuperPC	0.65229682	0.66297906	0.66216216
StepCox[both]+Ridge	0.65300353	0.60766495	0.64791155
StepCox[backward]+Ridge	0.67915194	0.58988542	0.62358722
StepCox[both]+plsRcox	0.56678445	0.59344133	0.63857494
StepCox[backward]+plsRcox	0.6819788	0.59245358	0.60933661
StepCox[both]+Enet[alpha=0.9]	0.58374558	0.58593441	0.63095823
StepCox[backward]+Enet[alpha=0.9]	0.58869258	0.56469775	0.61302211
StepCox[both]+Enet[alpha=0.1]	0.61342756	0.59719478	0.63734644
StepCox[backward]+Enet[alpha=0.1]	0.63321555	0.57418017	0.61621622
StepCox[both]+Enet[alpha=0.8]	0.57597173	0.57971158	0.62678133
StepCox[backward]+Enet[alpha=0.8]	0.59787986	0.56795733	0.61670762
StepCox[both]+Enet[alpha=0.2]	0.68056537	0.61803635	0.62874693
StepCox[backward]+Enet[alpha=0.2]	0.62614841	0.57289609	0.61670762
StepCox[both]+Lasso	0.67349823	0.61309759	0.63857494
StepCox[backward]+Lasso	0.66289753	0.59215725	0.6036855
StepCox[both]+Enet[alpha=0.6]	0.59293286	0.58850257	0.63243243
StepCox[backward]+Enet[alpha=0.6]	0.66219081	0.59334255	0.6031941
CoxBoost+GBM	0.56466431	0.59852825	0.55208845
StepCox[both]+Enet[alpha=0.7]	0.58303887	0.58583564	0.63071253
StepCox[backward]+Enet[alpha=0.7]	0.61272085	0.57101936	0.61498771
Lasso+StepCox[backward]	0.64452297	0.57822995	0.5958231
StepCox[both]	0.57102473	0.5776373	0.62530713
StepCox[backward]	0.57526502	0.56005531	0.61130221
StepCox[both]+Enet[alpha=0.4]	0.68056537	0.61892533	0.62972973
StepCox[backward]+Enet[alpha=0.4]	0.67208481	0.59719478	0.60761671
StepCox[both]+Enet[alpha=0.3]	0.59787986	0.59215725	0.63439803
StepCox[backward]+Enet[alpha=0.3]	0.60848057	0.56894508	0.61769042
StepCox[both]+CoxBoost	0.58869258	0.58761359	0.63194103
StepCox[backward]+CoxBoost	0.6565371	0.59452785	0.60515971
StepCox[both]+Enet[alpha=0.5]	0.57597173	0.57931648	0.62628993
StepCox[backward]+Enet[alpha=0.5]	0.59929329	0.567661	0.61670762
CoxBoost+RSF	0.50636042	0.53333663	0.52125307
Lasso+GBM	0.56678445	0.58188463	0.55933661
GBM	0.61024735	0.59838009	0.55454545
StepCox[both]+survivalSVM	0.71024735	0.61675227	0.62997543
StepCox[backward]+survivalSVM	0.70176678	0.60559068	0.62088452
Lasso+RSF	0.51166078	0.53387989	0.51842752
StepCox[both]+GBM	0.53180212	0.55180759	0.54914005
StepCox[backward]+GBM	0.5360424	0.54686883	0.55528256
StepCox[both]+RSF	0.50777385	0.493629	0.50577396
StepCox[backward]+RSF	0.50989399	0.50029633	0.51670762
StepCox[both]+SuperPC	0.72084806	0.61250494	0.64201474
StepCox[backward]+SuperPC	0.71024735	0.60262742	0.63832924

To enhance the accuracy of overall survival (OS) prediction, this study constructed a nomogram prediction model based on Cox regression, integrating the CPM risk score with seven common clinical parameters (**Figure 8B**). In the TCGA cohort, patients with high risk had significantly lower OS, and this result was validated in three additional validation cohorts (*p* < 0.001; **Figure 8C**). The ROC curve demonstrated stable predictive performance for 1, 3 and 5 year survival (AUC>0.75; **Figure 8D**). The Calibration Curve confirmed a high degree of consistency between predicted and actual survival rates in the prognostic cohort, indicating reliable model calibration (**Figure 8E**). In the feature importance analysis of the prognostic model, all 13 genes showed positive correlations with risk scores (**Supplementary Figure S9B**), with LACTB2 demonstrating the strongest predictive value. This analysis confirms that the predictive capabilities of both models are based on feature contributions consistent with the disease’s pathological mechanisms.

### 3.8 Predicting sensitive drugs for GC patients with LACTB2 overexpression

**Figure 9:**
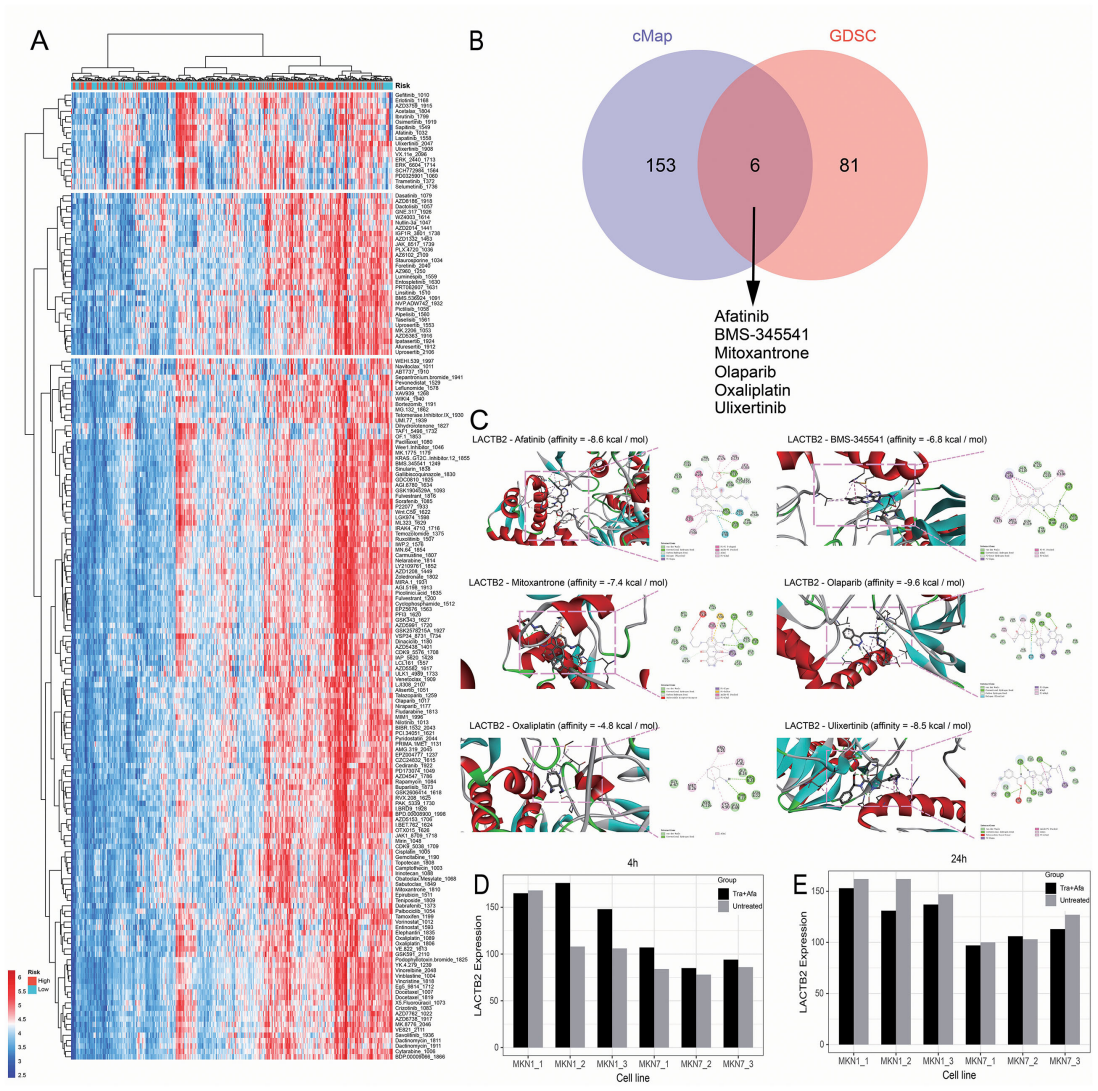
Potential inhibitors targeting LACTB2 overexpression: molecular docking and experimental validation. **(A)** Heatmap showing the different drug prediction scores based on machine learning. **(B)** The intersection of drugs with inhibitory and sensitive properties targeting LACTB2 overexpression. **(C)** Molecular docking to assess the binding ability of six overlapping drugs to the LACTB2 protein. **(D, E)** Trends in LACTB2 expression after co-treatment with Tra and Afa.

Based on the analysis of 179 drugs using the “oncoPredict” package, LACTB2 expression in GC patients was found to be associated with the IC50 of 87 drugs (**Figure 9A**; **Table 8**). To identify potential compounds targeting LACTB2-overexpressing GC, connectivity map (cMap) analysis, which was based on gene expression perturbation, identified 159 potential inhibitors (Score<-60; **Table 9**). By taking the intersection, six overlapping drugs were finally obtained (**Figure 9B**). Furthermore, the IC50 values of Ulixertinib, Afatinib, Oxaliplatin, and BMS-345541 were lower in the high LACTB2 expression group (*p* < 0.05; **Supplementary Figure S8**). Molecular docking between these drugs and the LACTB2 protein showed that LACTB2 had strong molecular affinities with Ulixertinib (affinity=-8.5kcal/mol), Afatinib (affinity=-8.6kcal/mol), and Olaparib (affinity=-9.6kcal/mol) (**Figure 9C**).

**Table 8:** Sensitivity of 87 drugs targeting LACTB2 overexpression in GC patients.

Drug	Avg1	Avg2	*p*	FC	Log_2_FC
WZ4003_1614	51.44636196	40.1024812	4.60E-08	0.779500817	-0.359377559
Dihydrorotenone_1827	2.338297076	3.028809492	5.09E-07	1.295305683	0.373292604
OF-1_1853	59.32199517	70.16064702	3.67E-06	1.182708822	0.242094932
JAK_8517_1739	28.8684715	21.03098588	5.04E-06	0.728510544	-0.456978242
IGF1R_3801_1738	7.362363145	5.230343075	8.15E-06	0.71041634	-0.493263331
GNE-317_1926	2.070517998	1.695477508	8.75E-06	0.818866346	-0.288300097
Acetalax_1804	142.4424717	219.0918188	9.02E-06	1.538107392	0.621156237
Gefitinib_1010	24.48024561	29.45692481	1.26E-05	1.203293679	0.266988794
AZD3759_1915	13.99575394	16.41920272	1.42E-05	1.173156001	0.230394869
SCH772984_1564	12.73895262	13.61727688	1.52E-05	1.068947918	0.096191563
Dasatinib_1079	7.742641884	5.874163752	2.64E-05	0.758676927	-0.398442432
Lapatinib_1558	21.10117635	28.25540027	2.69E-05	1.339043843	0.421203198
AZD8186_1918	33.81152983	27.49493308	2.84E-05	0.813182166	-0.298349519
Ulixertinib_2047	8.525608782	10.35431414	3.09E-05	1.214495575	0.280357234
ML323_1629	85.21732856	104.81938	3.71E-05	1.230024243	0.29868675
Afatinib_1032	6.206134784	7.957856185	5.64E-05	1.282256422	0.358684797
LGK974_1598	54.58814038	63.20780735	6.63E-05	1.157903657	0.211515219
TAF1_5496_1732	48.79413251	60.63742697	6.81E-05	1.242719644	0.313500863
AZD2014_1441	9.138363973	7.61176475	8.62E-05	0.832946113	-0.263704931
Ulixertinib_1908	15.22491438	18.99452306	0.000161349	1.247594737	0.319149372
Gallibiscoquinazole_1830	13.37036715	15.47562417	0.000177481	1.157456935	0.210958516
Osimertinib_1919	5.781098396	7.080068612	0.000193651	1.224692632	0.292419714
Sapitinib_1549	51.65482827	67.88079893	0.000201105	1.31412302	0.394100338
5-Fluorouracil_1073	128.7365929	183.3802467	0.000208828	1.424460929	0.510416051
AZD1332_1463	57.92469192	47.40702619	0.000391097	0.818425176	-0.289077569
AZ960_1250	10.32999412	8.420744879	0.000517256	0.815174218	-0.294819672
VSP34_8731_1734	10.49761664	12.3457996	0.000608487	1.176057388	0.233958461
Ibrutinib_1799	91.39401921	116.7104533	0.000950376	1.277003181	0.352762119
Oxaliplatin_1089	46.12772427	57.80733718	0.001010241	1.253201585	0.3256185
Carmustine_1807	443.9776304	506.2318671	0.001013667	1.140219309	0.189311338
Sinularin_1838	36.26671516	41.50671961	0.001055627	1.144485224	0.194698837
BMS-345541_1249	30.04182358	38.01399486	0.001084488	1.265369086	0.339558256
VX-11e_2096	16.76613191	20.74488511	0.001091816	1.237308953	0.307205782
Nutlin-3a (-)_1047	180.7839763	129.1892207	0.001219537	0.714605483	-0.484781111
Crizotinib_1083	25.45821722	30.49299326	0.001356439	1.197766246	0.260346381
VE821_2111	64.4416475	81.11341623	0.001402044	1.258711088	0.331947179
Fulvestrant_1816	93.29808326	104.8486675	0.002221862	1.123803017	0.168389179
Navitoclax_1011	7.791202789	9.333516714	0.002250309	1.197955818	0.260574701
Olaparib_1017	88.40408619	75.60765779	0.002443497	0.855250714	-0.225580692
Epirubicin_1511	0.452510428	0.354346695	0.002939413	0.783068572	-0.352789447
Dactolisib_1057	0.247698926	0.212761602	0.003050888	0.858952463	-0.219349805
PD0325901_1060	1.660250949	2.080238937	0.003050888	1.25296657	0.325347924
GSK1904529A_1093	73.49773235	84.12074436	0.003275388	1.144535235	0.194761877
CDK9_5038_1709	0.12841535	0.101888813	0.003305755	0.793431723	-0.333822015
P22077_1933	90.13865108	109.1816158	0.004054357	1.211263032	0.276512188
Mitoxantrone_1810	2.922834033	2.133842204	0.004115968	0.730059312	-0.453914417
AZD4547_1786	20.36432198	17.98267041	0.004128392	0.883047834	-0.179436505
PRIMA-1MET_1131	120.1830738	101.2193437	0.004293025	0.842209644	-0.2477487
AGI-6780_1634	61.41627312	66.2932862	0.00570512	1.079409134	0.110241801
PLX-4720_1036	100.8956486	92.11238179	0.005891172	0.912947021	-0.131396953
PRT062607_1631	30.00436963	26.37983481	0.006316214	0.879199768	-0.18573709
Uprosertib_1553	24.69663783	20.56735928	0.006576716	0.832799971	-0.263958076
Erlotinib_1168	13.15782821	15.23767501	0.006729874	1.158069156	0.211721408
GDC0810_1925	136.3193514	151.6792436	0.006985604	1.112675802	0.154033299
Temozolomide_1375	408.4075484	462.5983193	0.007480749	1.132687976	0.179750493
Pictilisib_1058	4.988588648	4.366406877	0.008189837	0.875278999	-0.19218514
Picolinici-acid_1635	167.1922141	180.7122082	0.008352747	1.080864975	0.112186308
Sabutoclax_1849	0.823375955	0.679491445	0.008376255	0.825250532	-0.277095931
Trametinib_1372	2.005689709	2.612312452	0.009084244	1.302450942	0.381229033
AZD5438_1401	9.741244368	11.31341423	0.010009533	1.161393124	0.215856398
Foretinib_2040	3.101798478	2.787950143	0.01020436	0.898817303	-0.153900196
XAV939_1268	86.42994899	80.97151163	0.011230017	0.936845533	-0.094116899
IRAK4_4710_1716	140.2490884	152.9644836	0.011322051	1.090662944	0.125205323
VE-822_1613	28.09018262	33.45954049	0.012853934	1.191147133	0.252351629
KRAS (G12C) Inhibitor-12_1855	84.96083206	98.73160547	0.013344384	1.162083787	0.216714092
Alisertib_1051	9.772585289	7.694775092	0.015114	0.787383775	-0.344861111
GSK591_2110	98.33981061	106.6531519	0.01543497	1.084536886	0.117079121
AZD5991_1720	96.62215944	120.9370603	0.016434153	1.251649321	0.323830414
Pyridostatin_2044	31.99995486	29.29367535	0.016649328	0.915428646	-0.127480656
MK-1775_1179	1.900669663	2.362836016	0.016779616	1.243159746	0.314011695
Nelarabine_1814	418.1144076	470.8430035	0.018179224	1.12611045	0.171348335
IAP_5620_1428	177.6324531	200.4294368	0.019184477	1.128337944	0.174199228
Selumetinib_1736	70.52781857	81.47951512	0.019730178	1.155281941	0.208244977
PCI-34051_1621	101.7659613	92.61674077	0.020704721	0.910095473	-0.135910196
Savolitinib_1936	13.0611649	14.2029214	0.023765991	1.087416131	0.120904134
Tamoxifen_1199	36.8950556	39.81933503	0.024788415	1.079259385	0.110041639
AZD6738_1917	8.653699708	10.48526815	0.025158258	1.211651491	0.276974794
Teniposide_1809	2.497149936	1.921016541	0.031606805	0.769283619	-0.378412506
MK-8776_2046	26.28904087	30.61926403	0.031834052	1.164715905	0.219978099
Irinotecan_1088	18.75832406	15.81158057	0.031910111	0.842910087	-0.246549348
JAK1_8709_1718	74.98715291	68.14916072	0.03702637	0.908811151	-0.137947559
LY2109761_1852	176.7126431	196.2892276	0.039333021	1.110782026	0.151575737
MN-64_1854	114.9398548	128.2971523	0.042145045	1.116211191	0.158610016
Luminespib_1559	0.12618889	0.128140728	0.048173255	1.015467596	0.022144204
Cytarabine_1006	6.309628238	7.475456832	0.04860693	1.184769776	0.244606742
LCL161_1557	140.5332136	153.5774205	0.048824995	1.092819388	0.128054985
Oxaliplatin_1806	165.9065663	182.2551137	0.048934334	1.09854069	0.135588308
NVP-ADW742_1932	18.17266024	16.65711861	0.048934334	0.916603205	-0.125630765

**Table 9:** Potential inhibitory compounds of 159 targeting LACTB2 overexpression in GC patients.

Rank	Score	Type	ID	Name	Description
7922	-60.02	cp	BRD-A77050075	heraclenol	Vitamin K antagonist
7924	-60.13	cp	BRD-A19248578	latrunculin-b	Actin polymerization inhibitor
7927	-60.21	cp	BRD-K05350981	oligomycin-c	ATPase inhibitor
7931	-60.43	cp	BRD-K98493452	honokiol	AKT inhibitor
7937	-60.65	cp	BRD-A94756469	digoxin	ATPase inhibitor
7940	-60.86	cp	BRD-K78659596	MLN-2238	Proteasome inhibitor
7946	-61.17	cp	BRD-A35989968	megestrol	Progesterone receptor agonist
7952	-61.27	cp	BRD-K67261995	adipiodone	Contrast agent
7954	-61.4	cp	BRD-A80638690	floxuridine	DNA synthesis inhibitor
7955	-61.41	cp	BRD-K68202742	trichostatin-a	HDAC inhibitor
7961	-61.64	cp	BRD-K49372556	mofezolac	Cyclooxygenase inhibitor
7962	-61.64	cp	BRD-A48430263	pioglitazone	Insulin sensitizer
7966	-61.81	cp	BRD-K94887716	TFMPP	Serotonin receptor agonist
7974	-62.09	cp	BRD-A66435872	HTMT	Histamine receptor agonist
7978	-62.14	cp	BRD-K88611939	aniracetam	Glutamate receptor agonist
7979	-62.16	cp	BRD-A64479082	quinidine	Sodium channel blocker
7991	-62.7	cp	BRD-K97365803	PI-828	PI3K inhibitor
7998	-62.91	cp	BRD-K11853856	PJ-34	PARP inhibitor
8000	-62.99	cp	BRD-K83972459	JWE-035	Aurora kinase inhibitor
8001	-63.16	cp	BRD-A37347161	BRL-52537	Opioid receptor agonist
8002	-63.17	cp	BRD-K37691127	hinokitiol	Tyrosinase inhibitor
8006	-63.47	cp	BRD-A45664787	iloprost	Platelet aggregation inhibitor
8008	-63.57	cp	BRD-K51290057	SA-792709	Retinoid receptor agonist
8013	-63.85	cp	BRD-K06753942	nobiletin	MEK inhibitor
8017	-64.1	cp	BRD-A39255369	DCPIB	Chloride channel blocker
8019	-64.15	cp	BRD-K06712146	YM-90709	IL5 inhibitor
8026	-64.62	cp	BRD-A62025033	temsirolimus	MTOR inhibitor
8028	-64.71	cp	BRD-K04887706	AKT-inhibitor-1-2	AKT inhibitor
8032	-64.86	cp	BRD-K27721098	clopidogrel	Purinergic receptor antagonist
8033	-64.88	cp	BRD-K94294671	OSI-027	MTOR inhibitor
8034	-64.93	cp	BRD-K52911425	GDC-0941	PI3K inhibitor
8039	-65.11	cp	BRD-K48115423	2-(4-methoxybenzylthio)-6-methylpyrimidin-4-ol	Matrix metalloprotease inhibitor
8040	-65.14	cp	BRD-K40175214	torin-1	MTOR inhibitor
8042	-65.28	cp	BRD-M40783228	mesna	Antioxidant
8049	-65.83	cp	BRD-A24381660	zeranol	Estrogen receptor agonist
8054	-66.24	cp	BRD-K11399644	phenformin	AMPK activator
8059	-66.45	cp	BRD-A56020723	CA-074-Me	Cathepsin inhibitor
8061	-66.5	cp	BRD-A71765365	mepireserpate	Catecholamine depleting sympatholytic
8076	-67.06	cp	BRD-U25771771	WZ-4-145	EGFR inhibitor
8088	-67.41	cp	BRD-K94689771	pinocembrin	CYP1B1 inhibitor
8096	-67.64	cp	BRD-K11558771	droxinostat	HDAC inhibitor
8098	-67.73	cp	BRD-K30097969	pitavastatin	HMGCR inhibitor
8099	-67.8	cp	BRD-K82164249	andarine	Androgen receptor modulator
8107	-68.2	cp	BRD-K14765469	vesamicol	Acetylcholinesterase inhibitor
8109	-68.23	cp	BRD-K67868012	PI-103	MTOR inhibitor
8111	-68.24	cp	BRD-A69512159	carbidopa	Aromatic L-amino acid decarboxylase inhibitor
8117	-68.58	cp	BRD-K08589866	linsitinib	IGF-1 inhibitor
8120	-68.75	cp	BRD-K93460210	lamotrigine	Serotonin receptor antagonist
8122	-68.81	cp	BRD-K41859756	NVP-AUY922	HSP inhibitor
8125	-69.06	cp	BRD-A28970875	puromycin	Protein synthesis inhibitor
8127	-69.38	cp	BRD-K81272440	dantrolene	Calcium channel blocker
8129	-69.63	cp	BRD-K58299615	RO-90-7501	Beta amyloid inhibitor
8132	-69.78	cp	BRD-A95513702	androsta-1,4-dien-3,17-dione	Aromatase inhibitor
8134	-69.94	cp	BRD-A65767837	hydrocortisone	Glucocorticoid receptor agonist
8141	-70.38	cp	BRD-K09499853	KU-0060648	DNA dependent protein kinase inhibitor
8200	-70.5	cp	BRD-K12867552	THM-I-94	HDAC inhibitor
8202	-70.55	cp	BRD-K37447567	hydrocotarnine	Opioid receptor antagonist
8204	-70.63	cp	BRD-K33312228	halometasone	Glucocorticoid receptor agonist
8210	-71.35	cp	BRD-A39415247	norethisterone	Progesterone receptor agonist
8215	-71.43	cp	BRD-K18518344	digitoxigenin	ATPase inhibitor
8219	-71.6	cp	BRD-K51967704	BIIB021	HSP inhibitor
8229	-71.91	cp	BRD-K87573634	propylpyrazole	Estrogen receptor agonist
8244	-72.87	cp	BRD-K17674993	diflorasone	Corticosteroid agonist
8258	-73.24	cp	BRD-A17535965	gelsemine	Acetylcholine receptor antagonist
8260	-73.28	cp	BRD-K07762753	aminopurvalanol-a	Tyrosine kinase inhibitor
8263	-73.54	cp	BRD-K22503835	scriptaid	HDAC inhibitor
8269	-73.78	cp	BRD-K17743125	belinostat	HDAC inhibitor
8272	-74.13	cp	BRD-K82147103	lofepramine	Norepinephrine reuptake inhibitor
8274	-74.17	cp	BRD-K99616396	motesanib	KIT inhibitor
8276	-74.21	cp	BRD-K22385716	LY-303511	Casein kinase inhibitor
8277	-74.23	cp	BRD-A36066264	estradiol-benzoate	Estrogen receptor agonist
8279	-74.44	cp	BRD-K86887724	dofetilide	Potassium channel blocker
8287	-74.81	cp	BRD-K64606589	apicidin	HDAC inhibitor
8291	-75.05	cp	BRD-K92870997	pterostilbene	Cyclooxygenase inhibitor
8293	-75.39	cp	BRD-A89434049	sarmentogenin	ATPase inhibitor
8297	-75.7	cp	BRD-K73290745	ICI-199441	Opioid receptor agonist
8304	-76.09	cp	BRD-A00758722	noretynodrel	Progestogen hormone
8307	-76.25	cp	BRD-A99833829	bethanechol	Acetylcholine receptor agonist
8308	-76.45	cp	BRD-K66175015	afatinib	EGFR inhibitor
8311	-76.55	cp	BRD-K16485616	mocetinostat	HDAC inhibitor
8315	-76.98	cp	BRD-K31633810	DAU-5884	Acetylcholine receptor antagonist
8319	-77.14	cp	BRD-A16478930	amcinonide	Glucocorticoid receptor agonist
8320	-77.16	cp	BRD-K15935639	z-leu3-VS	Proteasome inhibitor
8321	-77.25	cp	BRD-K39983086	loteprednol	Glucocorticoid receptor agonist
8326	-77.67	cp	BRD-A20697603	thiostrepton	FOXM1 inhibitor
8327	-77.67	cp	BRD-K68132782	terbinafine	Fungal squalene epoxidase inhibitor
8330	-77.84	cp	BRD-K06426971	ryuvidine	Histone lysine methyltransferase inhibitor
8340	-78.69	cp	BRD-K03109492	NSC-663284	CDC inhibitor
8351	-79.47	cp	BRD-K34508425	KUC103898N	
8355	-79.74	cp	BRD-K32398298	alprazolam	Benzodiazepine receptor agonist
8358	-80.09	cp	BRD-K15519488	CS-110266	Dopamine receptor agonist
8360	-80.31	cp	BRD-K12502280	TG-101348	FLT3 inhibitor
8361	-80.39	cp	BRD-K21283037	riluzole	Glutamate inhibitor
8362	-80.45	cp	BRD-K54704028	BAY-36-7620	Glutamate receptor antagonist
8363	-80.64	cp	BRD-K46056750	AZD-7762	CHK inhibitor
8371	-81.22	cp	BRD-K02113016	olaparib	PARP inhibitor
8381	-81.77	cp	BRD-K52522949	NCH-51	HDAC inhibitor
8386	-81.9	cp	BRD-A25067867	benzatropine	Acetylcholine receptor antagonist
8390	-82.15	cp	BRD-A45498368	WYE-125132	MTOR inhibitor
8397	-82.58	cp	BRD-K18855837	varenicline	Acetylcholine receptor agonist
8402	-82.86	cp	BRD-A79903587	tegafur	Thymidylate synthase inhibitor
8403	-82.87	cp	BRD-K43236057	piceid	ICAM1 inhibitor
8407	-83.06	cp	BRD-K70976396	cefoxitin	Bacterial cell wall synthesis inhibitor
8412	-83.69	cp	BRD-K69840642	ISOX	HDAC inhibitor
8414	-83.94	cp	BRD-A78360835	cercosporin	Photoactivated toxin
8422	-84.76	cp	BRD-K08554278	bisbenzimide	DNA binding agent
8428	-85.24	cp	BRD-K72222507	quinapril	ACE inhibitor
8429	-85.25	cp	BRD-K17953061	staurosporine	PKC inhibitor
8431	-85.29	cp	BRD-K92413528	thiazolidinecarboxylic-acid	Reducing agent
8437	-85.84	cp	BRD-K86958018	olvanil	TRPV agonist
8438	-85.84	cp	BRD-K63068307	ZSTK-474	PI3K inhibitor
8441	-86.33	cp	BRD-K98157055	SIB-1757	Glutamate receptor antagonist
8442	-86.35	cp	BRD-K60762818	desipramine	Tricyclic antidepressant
8446	-86.6	cp	BRD-A30437061	camptothecin	Topoisomerase inhibitor
8459	-87.53	cp	BRD-K18059238	gamma-linolenic-acid	Cyclooxygenase inhibitor
8462	-88.06	cp	BRD-A07765530	epinephrine	carbonic anhydrase activator
8465	-88.28	cp	BRD-K37194137	III606050	Cytochrome P450 inhibitor
8467	-88.41	cp	BRD-K89014967	AS-703026	MEK inhibitor
8472	-88.99	cp	BRD-K51677086	erythromycin	NFkB pathway inhibitor
8475	-89.44	cp	BRD-K41337261	ZM-306416	ABL inhibitor
8479	-89.77	cp	BRD-K24132293	piperlongumine	Glutathione transferase inhibitor
8480	-89.87	cp	BRD-K11927976	ER-27319	Mediator release inhibitor
8483	-90.03	cp	BRD-K48869804	icilin	TRPV agonist
8484	-90.12	cp	BRD-K13810148	givinostat	HDAC inhibitor
8485	-90.22	cp	BRD-K02130563	panobinostat	HDAC inhibitor
8487	-90.34	cp	BRD-A44448661	pentobarbital	Barbiturate antiepileptic
8488	-90.87	cp	BRD-K92093830	doxorubicin	Topoisomerase inhibitor
8489	-91.12	cp	BRD-K54233340	dorsomorphin	AMPK inhibitor
8491	-91.26	cp	BRD-K01436366	XMD-1150	Leucine rich repeat kinase inhibitor
8492	-91.26	cp	BRD-K13390322	AT-7519	CDK inhibitor
8494	-91.72	cp	BRD-K38615104	A-443644	AKT inhibitor
8500	-91.95	cp	BRD-K50387473	XMD-892	MAP kinase inhibitor
8502	-92.22	cp	BRD-K56001384	antimycin-a	ATP synthase inhibitor
8503	-92.54	cp	BRD-A18497530	5-iodotubercidin	Adenosine kinase inhibitor
8504	-92.57	cp	BRD-K21680192	mitoxantrone	Topoisomerase inhibitor
8508	-92.84	cp	BRD-A13122391	triptolide	RNA polymerase inhibitor
8509	-92.95	cp	BRD-K23192422	lestaurtinib	FLT3 inhibitor
8513	-93.58	cp	BRD-K44497846	enalapril	ACE inhibitor
8515	-93.71	cp	BRD-K64800655	PHA-793887	CDK inhibitor
8518	-93.94	cp	BRD-K43389675	daunorubicin	RNA synthesis inhibitor
8522	-94.59	cp	BRD-K69650333	idarubicin	Topoisomerase inhibitor
8524	-94.75	cp	BRD-A11702965	chromomycin-a3	DNA binding agent
8526	-94.79	cp	BRD-K96037667	norethindrone	Progesterone receptor agonist
8527	-94.82	cp	BRD-A59985574	topotecan	Topoisomerase inhibitor
8528	-94.86	cp	BRD-A73909368	dactinomycin	RNA polymerase inhibitor
8529	-95	cp	BRD-K06543683	bisindolylmaleimide-ix	CDK inhibitor
8532	-95.35	cp	BRD-K04548931	pidorubicine	Topoisomerase inhibitor
8542	-96.72	cp	BRD-K79090631	CGP-60474	CDK inhibitor
8544	-96.89	cp	BRD-K11636097	JNJ-7706621	CDK inhibitor
8545	-96.96	cp	BRD-A60245366	AS-601245	JNK inhibitor
8546	-96.97	cp	BRD-K19220233	JNK-9L	JNK inhibitor
8547	-96.99	cp	BRD-A88254928	salbutamol	Adrenergic receptor agonist
8548	-97.04	cp	BRD-A02333338	cyclopamine	Smoothened receptor antagonist
8549	-97.32	cp	BRD-K99545815	PF-562271	Focal adhesion kinase inhibitor
8550	-97.74	cp	BRD-K83794624	pirarubicin	Topoisomerase inhibitor
8551	-97.89	cp	BRD-K87909389	alvocidib	CDK inhibitor
8552	-97.92	cp	BRD-K13566078	BMS-345541	IKK inhibitor
8553	-98.03	cp	BRD-U51951544	ZG-10	JNK inhibitor
8554	-98.1	cp	BRD-M16762496	PIK-75	DNA protein kinase inhibitor

Trastuzumab combined with chemotherapy is a novel strategy for GC patients. MKN1 and MKN7 cells were grown in RPMI medium (10% FCS, 0.5% penicillin/streptomycin). After treatment with trastuzumab (Tra, 5 μg/ml, Roche) or afatinib (Afa, 0.5 μM, Biozol) for 4 or 24 hours, experiments were repeated three times. Compared to the untreated group, combined Tra and Afa treatment for 4 hours caused a transient increase in LACTB2 expression in GC cell lines (**Figure 9D**). When the treatment time was extended to 24 hours, LACTB2 expression decreased below the untreated group (**Figure 9E**). Six potential LACTB2 inhibitors were identified, with Afatinib and Ulixertinib possibly playing a big role in GC treatment.

### 3.9 Establishment of an early blood diagnosis model for GC based on LACTB2-upstream miRNA

**Figure 10:**
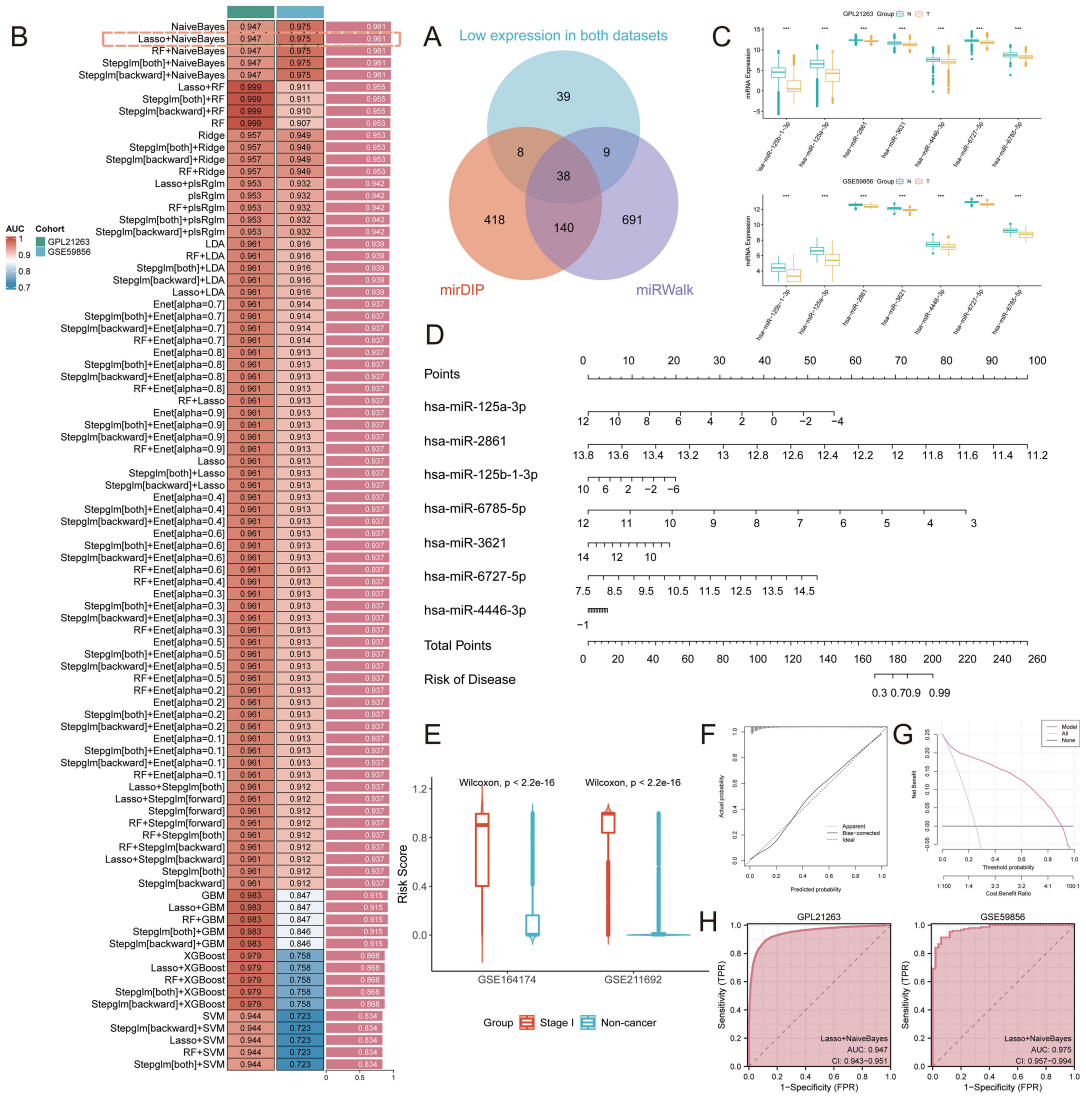
Construction of a model for the early diagnosis of GC based on LACTB2 upstream miRNA screening. **(A)** The miRNA screening process. **(B)** Construction of a miRNA diagnostic model based on multiple machine learning techniques. **(C)** Expression of seven miRNAs in GC blood samples. **(D)** A diagnostic column line graph constructed based on the expression of seven miRNAs. **(E)** The difference in risk scores between early GC blood samples and normal samples. **(F)** Calibration curves. **(G)** DCA validation. **(H)** ROC curve. ^ns/NS^p > 0.05, *p < 0.05, **p < 0.01, ***p < 0.001, ****p < 0.0001.

Based on two predictive miRNA tools (mirDIP and miRWalk), 38 GC-related low-expression miRNAs targeting LACTB2 in blood were identified (**Figure 10A**). Integrating 113 machine-learning methods and 13,505 GC blood samples, we selected the “Lasso + NaiveBayes” model as the CPM (**Figure 10B**; **Table 10**). The CPM included seven miRNAs (hsa-miR-125b-1-3p, hsa-miR-125a-3p, hsa-miR-2861, hsa-miR-3621, hsa-miR-4446-3p, hsa-miR-6727-5p, hsa-miR-6785-5p), which were highly expressed in two cohorts (**Figure 10C**). To enhance GC diagnosis, we built a diagnostic nomogram based on these seven miRNAs (**Figure 10D**).

**Table 10:** The performance of 113 predictive models in training and testing cohorts.

Model	GPL21263	GSE59856
Lasso+Stepglm[both]	0.96118365	0.912
SVM	0.944188604	0.723333333
Ridge	0.957307381	0.9488
Lasso+SVM	0.943960575	0.723333333
Enet[alpha=0.1]	0.96118295	0.912666667
Enet[alpha=0.2]	0.961181931	0.9128
Enet[alpha=0.3]	0.961184987	0.913066667
Enet[alpha=0.4]	0.961185782	0.9132
Enet[alpha=0.5]	0.961184223	0.913066667
Enet[alpha=0.6]	0.961185337	0.9132
Enet[alpha=0.8]	0.961188265	0.913466667
Enet[alpha=0.9]	0.961188328	0.913333333
Lasso	0.961187214	0.913333333
Enet[alpha=0.7]	0.961187183	0.9136
Lasso+plsRglm	0.952916209	0.9316
Lasso+Stepglm[forward]	0.96118365	0.912
RF+SVM	0.943960575	0.723333333
Stepglm[forward]	0.96118365	0.912
plsRglm	0.952916209	0.9316
RF+Ridge	0.957307317	0.9488
RF+Enet[alpha=0.1]	0.961181931	0.912666667
RF+plsRglm	0.952916209	0.9316
RF+Stepglm[forward]	0.96118365	0.912
RF+Enet[alpha=0.2]	0.961182504	0.9128
RF+Enet[alpha=0.3]	0.961184477	0.913066667
RF+Enet[alpha=0.6]	0.961185178	0.9132
RF+Lasso	0.961186164	0.913466667
RF+Enet[alpha=0.7]	0.961185719	0.9136
RF+Enet[alpha=0.5]	0.961183777	0.913066667
RF+Enet[alpha=0.9]	0.961187278	0.913333333
RF+Enet[alpha=0.4]	0.961184859	0.9132
RF+Enet[alpha=0.8]	0.961186292	0.913466667
RF+Stepglm[both]	0.96118365	0.912
RF+Stepglm[backward]	0.96118365	0.912
Stepglm[both]+Ridge	0.957307381	0.9488
Stepglm[backward]+Ridge	0.957307381	0.9488
Stepglm[both]+plsRglm	0.952916209	0.9316
Stepglm[backward]+plsRglm	0.952916209	0.9316
Stepglm[both]+Enet[alpha=0.9]	0.961188328	0.913333333
Stepglm[backward]+Enet[alpha=0.9]	0.961188328	0.913333333
Stepglm[both]+Enet[alpha=0.1]	0.96118295	0.912666667
Stepglm[backward]+Enet[alpha=0.1]	0.96118295	0.912666667
Stepglm[both]+Enet[alpha=0.8]	0.961188265	0.913466667
Stepglm[backward]+Enet[alpha=0.8]	0.961188265	0.913466667
Stepglm[both]+Enet[alpha=0.2]	0.961181931	0.9128
Stepglm[backward]+Enet[alpha=0.2]	0.961181931	0.9128
Stepglm[both]+Lasso	0.961187214	0.913333333
Stepglm[backward]+Lasso	0.961187214	0.913333333
Stepglm[both]+Enet[alpha=0.6]	0.961185337	0.9132
Stepglm[backward]+Enet[alpha=0.6]	0.961185337	0.9132
Stepglm[both]+Enet[alpha=0.7]	0.961187183	0.9136
Stepglm[backward]+Enet[alpha=0.7]	0.961187183	0.9136
Lasso+Stepglm[backward]	0.96118365	0.912
Stepglm[both]	0.96118365	0.912
Stepglm[backward]	0.96118365	0.912
Stepglm[both]+Enet[alpha=0.4]	0.961185782	0.9132
Stepglm[backward]+Enet[alpha=0.4]	0.961185782	0.9132
Stepglm[both]+Enet[alpha=0.3]	0.961184987	0.913066667
Stepglm[backward]+Enet[alpha=0.3]	0.961184987	0.913066667
Stepglm[both]+Enet[alpha=0.5]	0.961184223	0.913066667
Stepglm[backward]+Enet[alpha=0.5]	0.961184223	0.913066667
RF	0.999205002	0.9072
Lasso+GBM	0.983275247	0.846666667
RF+GBM	0.983279957	0.846533333
GBM	0.983292528	0.847066667
Stepglm[both]+SVM	0.943895412	0.723333333
Stepglm[backward]+SVM	0.944188604	0.723333333
Lasso+RF	0.999201692	0.911066667
Stepglm[both]+GBM	0.983268659	0.846266667
Stepglm[backward]+GBM	0.983316842	0.846
Stepglm[both]+RF	0.999201596	0.910533333
LDA	0.960968765	0.9164
RF+LDA	0.960968765	0.9164
Stepglm[both]+LDA	0.960968765	0.9164
Stepglm[backward]+LDA	0.960968765	0.9164
Lasso+LDA	0.960968765	0.9164
Stepglm[backward]+RF	0.99921665	0.909866667
XGBoost	0.978685484	0.758066667
Lasso+XGBoost	0.978685484	0.758066667
RF+XGBoost	0.978685484	0.758066667
Stepglm[both]+XGBoost	0.978685484	0.758066667
Stepglm[backward]+XGBoost	0.978685484	0.758066667
NaiveBayes	0.946608304	0.975333333
Lasso+NaiveBayes	0.946608304	0.975333333
RF+NaiveBayes	0.946608304	0.975333333
Stepglm[both]+NaiveBayes	0.946608304	0.975333333
Stepglm[backward]+NaiveBayes	0.946608304	0.975333333

Furthermore, among the 2,744 early GC (stage I) blood samples and 6,949 normal control samples collected, we observed that early GC patients exhibited significantly higher risk scores than normal samples, demonstrating the model’s capacity for early diagnosis (p < 0.0001; **Figure 10E**). Calibration curves and DCA validated the model’s reliability and accuracy (**Figures 10F, G**). The GPL21263 cohort (AUC = 0.947) and GSE59856 cohort (AUC = 0.975) ROC curves indicated the model had excellent diagnostic efficacy (**Figure 10H**). To assess the biological plausibility of the final model, we performed feature importance analysis. In the diagnostic model, expression levels of all 7 miRNAs showed significant negative correlations with risk scores (**Supplementary Figure S9A**), with hsa-miR-125a-3p exhibiting the strongest association with hsa-miR-125b-1-3p (Spearman correlation coefficients of -0.727 and -0.683, respectively). This aligns closely with the negative weights assigned by Lasso regression, consistent with their biological roles as upstream inhibitors of LACTB2.

## 4 Discussion

The predictive value of pan-LACTB2 mRNA overexpression for prognosis and treatment response in digestive tract tumours remains unexplored, especially in GC. This study screened 10,581 tissue samples for a comprehensive analysis of LACTB2 in digestive tract tumours and collected 236 internal clinical tissue samples for IHC experiments. The study integrated multi-omics data to identify dysregulated expression of LACTB2 in bulk RNA-seq, scRNA-seq, and spatial transcriptomics, highlighting the significant impact of LACTB2 overexpression on the progression of digestive tract tumours. Epigenetics provided upstream explanations for the causes of dysregulated expression. Although previous studies attempted to investigate the molecular mechanisms of LACTB2 in CRC, the opportunity for cross-digestive tract tumours research was lost due to the lack of sample size (56). This study also explored the prognosis and treatment response of LACTB2, using a prognostic model to predict risk stratification for GC patients at 1 year, 3 years, and 5 years. Additionally, the miRNA early screening model constructed from 13,505 GC blood samples in this study offers a less invasive, more convenient, and more valuable early diagnostic prediction for GC.

Overexpression of LACTB2 is closely associated with the development and metastasis of digestive tract tumours. Previous studies have found that LACTB2 can mediate the tumorigenesis and progression of CRC by regulating oxidative phosphorylation and the mTORC1 signalling pathway (56). Through clinical analysis, enrichment analysis, and immune analysis of a large number of digestive tract tumours samples, we explained the pro-cancer ability of LACTB2 across cancer types from multiple perspectives. Through ST analysis, we identified LACTB2 as a key molecule driving early malignant transformation in gastric cancer, specifically enriched in the tumour core region exhibiting the highest malignancy. Therefore, this study hypothesises that LACTB2 may establish a cross-cancer-type universal malignant proliferation-survival circuit, mediating immune suppression and immune evasion to exhibit a conserved oncogenic capacity across various cancers. Concurrently, tumour epithelial regions exhibiting high LACTB2 expression form spatially mutually exclusive patterns with immune cells, suggesting that LACTB2-overexpressing tumour cells may actively shape a proactive “immune exclusion” pattern. Quantitative analysis of spatial distance provides rigorous statistical support for the immune exclusion hypothesis from a spatial perspective. This finding provides a direct spatial interpretation for the immunosuppressive state observed via scRNA-seq. Furthermore, IHC experiments and the upstream transcriptional regulatory network of LACTB2 demonstrated the dysregulated expression of LACTB2 in GC and its potential regulatory mechanisms. This study integrated scRNA-seq and spatial transcriptomics to discover that LACTB2 may exert its pro-cancer effects by activating downstream pathways such as MAPK through the MK signalling pathway. This hypothesis is further supported by our single−cell perturbation analysis, in which virtual knockout of LACTB2 led to significant suppression of the MK signalling pathway in epithelial cells and reduced expression of key pathway genes. Therefore, we hypothesize that tumour cells overexpressing LACTB2 activate the MAPK pathway via paracrine MK signalling, thereby forming a signalling loop that drives malignant proliferation and survival.

As a tumour suppressor and diagnostic marker, miRNA plays a crucial role in cancer progression (57). In recent years, research focus has shifted towards developing early cancer diagnostic techniques based on miRNA in body fluid samples such as blood, saliva, and urine (58, 59). The diagnostic and prognostic models developed in this study demonstrate significant potential for clinical application. The diagnostic model, based on circulating blood-derived miRNAs, offers unique advantages of being non-invasive and convenient. It exhibits high sensitivity for early-stage gastric cancer (Stage I) and holds promise in addressing the limitations of existing serum biomarkers with insufficient sensitivity. The prognostic model effectively enables risk stratification, identifying high-risk patient groups with shorter overall survival who may benefit from intensified treatment. Furthermore, the integrated CPM risk score and clinical parameter nomogram provide a practical tool for personalized prognosis assessment and treatment decision-making. However, the model relies on retrospective data from public databases, introducing potential selection bias. Its generalizability requires further validation in prospective, multicenter cohorts. Second, current models rely on high-throughput sequencing data. Future clinical implementation requires establishing standardized testing protocols and defining consistent thresholds. Furthermore, the cost-effectiveness of multi-molecular biomarker combinations in routine clinical practice warrants further evaluation. Additionally, although Li et al. have previously explored quercetin components targeting LACTB2, this study comprehensively investigates potential inhibitory compounds and sensitive drugs targeting LACTB2 overexpression through machine learning prediction (56). The observed transient upregulation of LACTB2 following 4 hours of combined Trastuzumab and Afatinib treatment, followed by its subsequent decline, may reflect an abortive adaptive stress response. We speculate that cancer cells rapidly induce LACTB2 as part of a failed compensatory attempt to maintain survival signalling upon acute therapeutic insult, prior to the eventual collapse of pro-survival pathways under sustained drug exposure.

However, certain limitations are unavoidable. Further cell-based and animal-based experiments are required to validate the therapeutic potential of Afatinib and Ulixertinib, as well as to confirm the proposed oncogenic mechanisms of LACTB2. In addition, further validation in prospective cohorts and real-world clinical settings is required to optimize and establish the early diagnosis model for GC.

## 5 Conclusion

This study integrates a large number of multicentre samples and highlights the pro-oncogenic role of overexpressed LACTB2 in digestive tract tumours. LACTB2 may serve as a prognostic marker and a potential therapeutic target for GC, and its upstream miRNAs possess the potential for early diagnosis of GC in the blood.

## Data Availability

The original contributions presented in the study are included in the article/**Supplementary Material**. Further inquiries can be directed to the corresponding authors.

## Supplementary material

Supplementary Figure S1 Flow of digestive tract tumours related dataset screening in this study. **(A)** CRC tissue mRNA samples. **(B)** ESCA tissue mRNA samples. **(C)** GC tissue mRNA samples. **(D)** GC blood miRNA samples.

Supplementary Figure S2 A comparison of the difference in LACTB2 mRNA expression between the CRC and control groups. **(A)** Forest plot showing the standardised mean difference (SMD) for LACTB2 mRNA in CRC tissues. **(B)** Egger’s test. **(C)** Sensitivity analysis of LACTB2 mRNA expression (full dataset). **(D)** Sensitivity analysis of LACTB2 mRNA expression (with GPL570 and TCGA_GTEx removed). **(E)** ROC curves for each dataset. **(F)** sROC curves.

Supplementary Figure S3 A comparison of the difference in LACTB2 mRNA expression between the ESCA and control groups. **(A)** Forest plot showing the standardised mean difference (SMD) for LACTB2 mRNA in CRC tissues. **(B)** Egger’s test. **(C)** Sensitivity analysis of LACTB2 mRNA expression (full dataset). **(D)** Sensitivity analysis of LACTB2 mRNA expression (with GPL570 and TCGA_GTEx removed). **(E)** ROC curves for each dataset. **(F)** sROC curves.

Supplementary Figure S4 A comparison of the difference in LACTB2 mRNA expression between the GC and control groups. **(A)** Forest plot showing the standardised mean difference (SMD) for LACTB2 mRNA in CRC tissues. **(B)** Egger’s test. **(C)** Sensitivity analysis of LACTB2 mRNA expression (full dataset). **(D)** Sensitivity analysis of LACTB2 mRNA expression (with GPL570 and TCGA_GTEx removed). **(E)** ROC curves for each dataset. **(F)** sROC curves.

Supplementary Figure S5 Immunocorrelation analysis of LACTB2 expression and digestive tract tumours tumours. **(A–C)** Assessment of immune infiltration abundance in GI tumours based on three algorithms: EPIC, MCPcounter and QUANTIseq. **(D)** ESTIMATE-based analysis of the correlation between LACTB2 expression and the immune microenvironment in colon cancer. **(E)** An ESTIMATE-based analysis of the correlation between LACTB2 expression and the immune microenvironment in ESCA. **(F)** Correlation of LACTB2 expression with the immune microenvironment in rectal cancer based on ESTIMATE analysis.

Supplementary Figure S6 Analysis of LACTB2 in GC based on scRNA-seq. **(A)** Differentiation potential of different cell clusters. **(B)** UMAP plot of LACTB2 expression in different cells, generated using the “monocle3” package. **(C)** “CytoTRACE” was used to determine the viability of cell cluster differentiation and LACTB2 expression. **(D)** Communication relationships between the L-R pair of the MK signalling pathway in different cell clusters. **(E)** Afferent/efferent global communication patterns of cell clusters.

Supplementary Figure S7 An integrated, spatial transcriptomics-based analysis of LACTB2 expression in GC. **(A)** Chromosome-specific CNV signalling fluctuations. **(B)** Distribution of different cell types in spatial regions. **(C)** Expression of key MK signalling pathway genes in different regions. **(E)** Distribution of key MK signalling pathway genes in spatial regions.

Supplementary Figure S8 Differential analysis of LACTB2 expression and the IC50 values of six drugs.

Supplementary Figure S9 **(A)** Feature importance analysis demonstrating the significance of each miRNA within the diagnostic model. **(B)** Feature importance analysis demonstrating the significance of each gene within the prognostic model. **(C)** TIDE scoring evaluates the association between LACTB2 expression and tumour immune evasion potential in gastric cancer. **(D)** Correlation analysis of LACTB2 expression with its upstream transcription factor FOXA1 in tumour versus control groups.

Supplementary Figure S10 **(A)** Intercellular communication strength of the MK signalling pathway, comparing Control (blue) and LACTB2 virtual knockout (KO, red) conditions. The plot is split into Incoming (top) and Outgoing (bottom) communication probabilities across distinct cell types in the tumour microenvironment. Following LACTB2 KO, the intensity of intercellular communication via the MK signalling pathway in epithelial cells decreases. **(B)** Violin plots showing normalised expression of MK pathway genes in epithelial cells between Control and LACTB2 KO groups. Significance was determined by Wilcoxon test (ns: not significant; p < 0.0001 indicated). The expression levels of key genes in the MK pathway in epithelial cells were significantly downregulated following LACTB2 KO. **(C)** Intercellular communication network of the MK signalling pathway following LACTB2 KO, visually demonstrating the sender, receiver, mediator and influencer roles of each cell type. Colour intensity reflects the strength of communication flow between distinct cell populations. In epithelial cells following LACTB2 KO, intercellular communication involving various components of the MK signalling pathway is impaired (compared with **Figure 5K**). **(D)** Spatial separation between the core regions of tumours expressing high levels of LACTB2 and immune cells.
